# Overall electrochemical splitting of water at the heterogeneous interface of nickel and iron oxide

**DOI:** 10.1038/s41467-019-13415-8

**Published:** 2019-12-06

**Authors:** Bryan H. R. Suryanto, Yun Wang, Rosalie K. Hocking, William Adamson, Chuan Zhao

**Affiliations:** 1 School of Chemistry, The University of New South Wales, Kensington, NSW 2052 Australia; 2 Centre for Clean Environment and Energy, School of Environment and Science, Griffith University, Gold Coast, QLD 4222 Australia; 3 Department of Chemistry and Biotechnology, Swinburne University of Technology, Hawthorn, Melbourne, VIC 3122 Australia

**Keywords:** Electrocatalysis, Nanoparticles

## Abstract

Efficient generation of hydrogen from water-splitting is an underpinning chemistry to realize the hydrogen economy. Low cost, transition metals such as nickel and iron-based oxides/hydroxides have been regarded as promising catalysts for the oxygen evolution reaction in alkaline media with overpotentials as low as ~200 mV to achieve 10 mA cm^−2^, however, they are generally unsuitable for the hydrogen evolution reaction. Herein, we show a Janus nanoparticle catalyst with a nickel–iron oxide interface and multi-site functionality for a highly efficient hydrogen evolution reaction with a comparable performance to the benchmark platinum on carbon catalyst. Density functional theory calculations reveal that the hydrogen evolution reaction catalytic activity of the nanoparticle is induced by the strong electronic coupling effect between the iron oxide and the nickel at the interface. Remarkably, the catalyst also exhibits extraordinary oxygen evolution reaction activity, enabling an active and stable bi-functional catalyst for whole cell water-splitting with, to the best of our knowledge, the highest energy efficiency (83.7%) reported to date.

## Abstract (web summary)

Ni–Fe based compound are known as active electrocatalysts for oxygen evolution reaction, but not a good choice for the other half-reaction of water-splitting. Here the authors report a unique interface between Ni and γ-Fe_2_O_3_ that efficiently catalyzes the cathodic hydrogen evolution reaction.

## Introduction

Electrochemical water-splitting has been considered as one of the most promising approaches to store renewable electricity in the form of hydrogen fuel^1^. Hydrogen can be generated in a water electrolyzer consisting of a hydrogen evolution reaction (HER) cathode and an oxygen evolution reaction (OER) anode^2,3^. However, due to the distinctly different catalytic mechanisms, active HER catalysts are often found to be poor OER catalysts, and vice versa^4,5^. The current benchmark electrolyzer utilizes Pt-based cathode and RuO_2_/IrO_2_ anodes to expedite HER and OER, respectively^6,7^. From a commercialization point-of-view, it is not only the significant cost of noble metal elements that creates economic pressure but also the additional cost generated due to the complications of producing different cathode–anode materials and possible cross-contaminations^8^. Hence the development of a universally active water-splitting catalyst based on Earth-abundant materials is of key interest and a significant innovation^9^.

Recent progress in the development of Earth-abundant-based electrode materials for OER has revealed that Ni and Fe mixed oxides/hydroxides system is inherently active in alkaline media, comparable to that of RuO_2_/IrO_2_-based electrocatalysts^10–12^. To this end, several strategies such as nanosizing, heteroatoms-doping, surface engineering, vacancy engineering, and carbon nanomaterials coupling have been demonstrated to improve the OER catalytic performance^11,13–16^. However, Ni–Fe-based alloys and oxide/hydroxide are not good cathode catalysts for HER, exhibiting sluggish kinetics at the oxides surface^17,18^. It is known that most of the Earth-abundant transition metals (M=Ni, Co, Fe, Mo, etc.) bind to H^+^ either too strongly or too weakly which results in poor HER activities^19^. As a result, Pt-based catalysts (e.g. Pt/C) remain the primary cathode material option to achieve extremely low overpotentials and large current densities^20^.

Significant recent effort has been made in the development of Earth-abundant transition metal chalcogenides and pnictogenides that exhibit bi-functional activity towards both HER and OER^21–26^. It is established that the modification of transition metal with electronegative chalcogens/pnictogens (X=N, P, S, and Se) can generate localized negative charges within the M–X surface structures which favors the initial adsorption of H^+^ (acidic) or H_2_O (alkaline), and near-surface electronic structure modulation, resulting in weakened M–H bond and lowered HER energy barriers^27–30^. However, OER at these materials normally occurs at the high valence metal active sites formed in operando during anodic polarization^31,32^. This anodic process inevitably leads to oxidative corrosion and restructuring of the M–X materials^32–34^. Concomitantly, the HER catalytic activity of the M–X materials degrades eventually due to the corrosion-induced compositional and structural changes. This issue is exacerbated if the electrolyzer is powered by intermittent renewable energy such as solar or wind, where electrode degradation is accelerated as a result of depolarization and reversed current induced by frequent power interruptions and shut-downs^35^.

Hence, the development of low cost and robust catalysts with intrinsic active sites that are capable of catalyzing distinctly different HER and OER processes is highly challenging but imperative to realize efficient and sustained intermittent water-splitting. A common method of achieving bi-functional catalyst is through the design of catalysts that exhibit multiple active sites or multi-site functionalities. One approach is the adoption of Janus particle design, a term coined Casagrande et al. in ref. ^36^, to describe an asymmetric particle which surface exhibit distinct composition/physical properties and therefore exhibits a distinct interface. Furthermore, it is also widely accepted that the creation of strongly coupled interface could benefits electrocatalysis due to the modulated electronic structure of the material^37^.

**Fig. 1:**
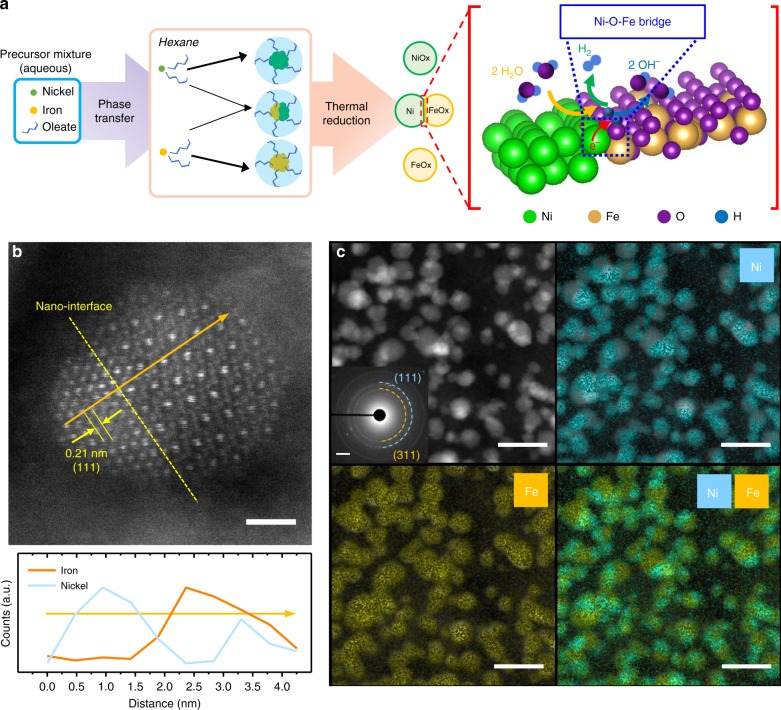
Nanoparticle design and electron microscopies. **a** Schematic representation of the Ni and Fe nanoparticles and the Ni-Fe Janus nanoparticles synthesis through the oleate-assisted micelle formation and the illustration on the HER across the Ni-γ-Fe_2_O_3_ interface in alkaline medium. **b** STEM-HAADF image of a single Ni–Fe NP nanoparticle and its corresponding EDS line-scan spectrum (scale bar: 1 nm). **c** High-resolution EDS mapping on STEM-HAADF images of the nanoparticles for Ni and Fe, selected area electron diffraction inset (image scale bars: 20 nm; SAED scale bar: 2 nm^−1^).

Herein, we show a Janus Ni–Fe nanoparticle (denoted Ni–Fe NP) exhibiting a Ni metal domain interconnected to a γ-Fe_2_O_3_, creating a heterojunction/interface (Fig. 1). Density functional theory (DFT) calculations indicate that the Ni–O–Fe bridge at Ni-γ-Fe_2_O_3_ interface modifies the Gibbs free energy of the adsorption of the intermediate H atoms (Δ*G*_H*_), thereby further promoting the performance of HER catalysis. As a result, the Ni–Fe NPs exhibit extraordinary HER catalytic activity, comparable to that of the benchmark Pt/C catalyst. Intriguingly, the overpotential for OER is also lowered due to the multi-site functionalities created at the interface as previously proposed by Nørskov and co-workers^38^. Through the combination of physical and electrochemical characterization methods, we have demonstrated that the Ni–Fe NPs exhibit extraordinary physical and electrochemical stability for both HER and OER processes.

## Results

### Physical characterizations

Important to this catalyst design is the synthesis of monodispersed Ni–Fe nanoparticles with controlled composition and very small size so that iron oxides (γ-Fe_2_O_3_) are formed adjacent to metallic Ni, and abundant and stable interfaces are available for catalyzing water-splitting processes. In this study, Ni–Fe NPs were prepared by initially synthesizing Ni, Fe ion-oleate metal-surfactant complexes. Subsequently, micelle formation by the complexes was induced by the use of non-polar solvent (*n*-hexane), serving as templates for nanoparticle formation during the thermal reduction process as illustrated in Supplementary Fig. 1. This approach is scalable and enables precise control over the composition and size of the nanoparticles (details in Methods)^39^.

The transmission electron microscopy (TEM) images (Supplementary Fig. 5) display the structure and size distribution of the as-synthesized Ni–Fe NPs, which exhibit a Gaussian profile with the majority of particles having diameters of 5–8 nm. Supplementary Figure 5a also reveals that the ion-oleate surfactants are carbonized to form conductive carbon network that may facilitate facile electron transfer between the nanoparticle and the support^8^. Utilizing a high angle annular dark-field (HAADF) STEM technique, Fig. 1b reveals the presence of interfaces which divide the Ni–Fe NP into two phases with distinctly different crystal structures and electron densities. The phase with higher electron density appears brighter in Fig. 1b, which reveals a closely packed Ni crystal structure. The darker phase exhibits a more sparsely packed, less-dense crystal structure filled with smaller oxygen atoms consistent with the γ-Fe_2_O_3_ structure, which was verified with the EDS line-scan elemental profiling shown in Fig. 1b. The EDS line scan indicates a distinct cross-over between the Ni and Fe elemental counts, confirming the presence of the Ni-γ-Fe_2_O_3_ heterojunction. The slightly increased Ni counts within the Fe-rich phase could be ascribed to the presence of another Ni-rich phase or overlapping nanoparticle. Lower-magnification EDS elemental mapping shows that the asymmetric distribution of Ni and Fe are also commonly observed in other particles. Importantly, the HR-STEM imaging reveals the formation of Ni–O–Fe bridge at the interface as highlighted in Supplementary Fig. 6. The selected area electron diffraction (SAED) of Ni–Fe NPs in Fig. 1c displays two clear diffraction rings with radius of 2.51 and 2.10 Å, indexed for (311) and (111) in γ-Fe_2_O_3_ and Ni crystal lattices, respectively. To understand the Ni–Fe NP formation mechanism, Ni- and Fe-based nanoparticles (denoted as Ni NPs and Fe NPs) were also synthesized via the identical method. The micrograph in Supplementary Fig. 7 shows that the Fe NPs are evenly distributed, exhibiting average particle diameters of 6–7 nm, similar to Ni–Fe NPs. On the other hand, the Ni NPs display an uneven distribution of large nanoparticles (~50 nm diameter, Supplementary Fig. 8). The nanoparticle size difference suggests that the thermal nucleation of Fe NPs occurs at lower temperatures (~70 °C) and is instantaneous, while the Ni NPs nucleate at higher temperature following a progressive mechanism^40,41^. These results suggest that the Ni–Fe NPs are formed by growth of Ni on the pre-formed Fe NP, rather than simultaneous nucleation.

**Fig. 2:**
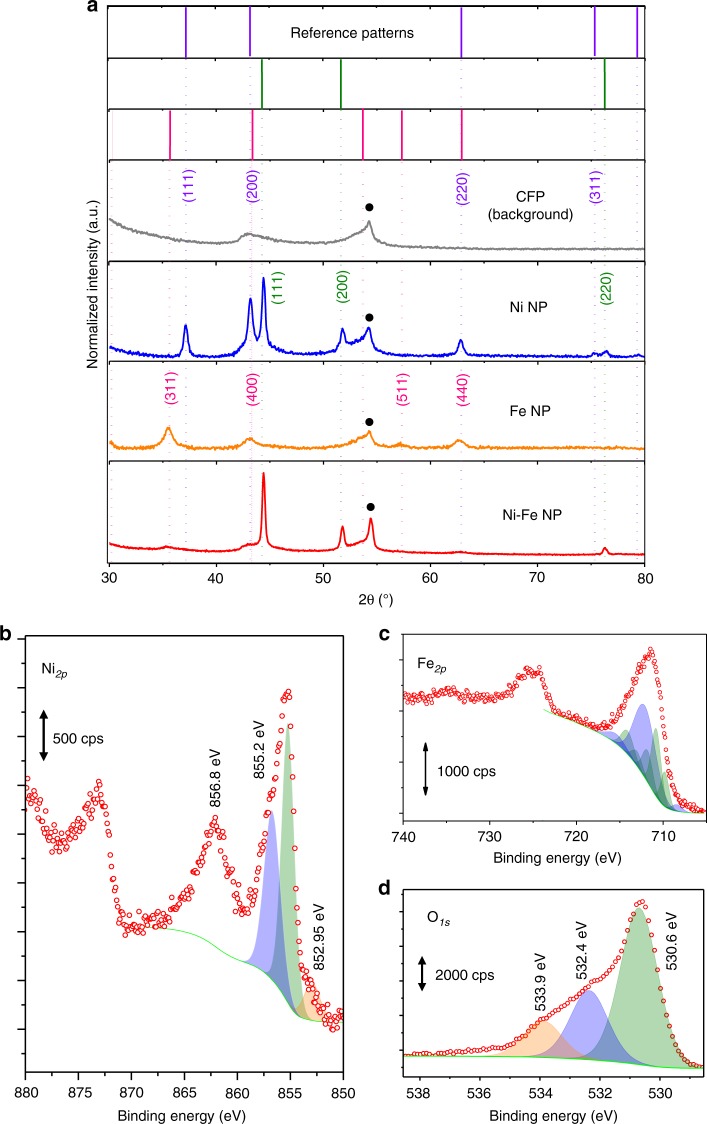
X-ray characterizations. **a** X-ray diffraction spectra showing the reference stick patterns of NiO (purple line, ICDD: 04-011-9039), Ni (green line, ICDD: 04-001-3331), and γ-Fe_2_O_3_ (magenta line, ICDD:01-078-6916). The peaks marked with black dots correspond to CFP substrate; X-ray photoelectron spectroscopy (XPS) of Ni–Fe NP. **b** Ni 2*p*, **c** Fe 2*p*, **d** O 1*s*.

X-ray diffraction (XRD) analysis shown in Fig. 2a also reveals the unique features of Ni–Fe NPs. XRD pattern of Ni NP indicates the formation of core-shell structure of Ni/NiO as identified by the presence of peaks correspond to both Ni and NiO (ICDD: 04-001-3331; 04-011-9039), as a result of instantaneous formation of passivating NiO outer shell^42^. The XRD of Fe NP indicates that γ-Fe_2_O_3_ (ICDD: 01-078-6916) was formed. In contrast, the nickel oxide diffraction peak was not observed in all the Ni–Fe NP samples and the strong peak at 44.5° corresponds to the (111) facet of metallic Ni, consistent to the STEM characterizations. Furthermore, the presence of γ-Fe_2_O_3_ in the Ni–Fe NP sample was confirmed by the identification of a set of diffraction peaks that match the Fe NP control material.

The oxidation states of Ni and Fe in Ni–Fe NPs were further analyzed by X-ray photoelectron spectroscopy (XPS). Two main peaks related to Ni 2*p* exist at ~855.2 eV (Ni 2*p*_3/2_) and 873.0 eV (Ni 2*p*_1/2_) in Ni–Fe NPs as shown in Fig. 2b are related to the unintended formation of large Ni/NiO NP (~50 nm in diameter) present in the Ni–Fe NP samples, as shown in Supplementary Fig. 10a. Further quantification based on area analysis of Supplementary Fig. 10b reveals that there are approximately 17% of NiO NP present in the Ni–Fe NP sample. The Ni 2*p*_3/2_ peak at 852.9 eV that corresponds to Ni^0^ (ref. ^43^) is observed only for the Ni–Fe NPs but not for the Ni NPs (Supplementary Fig. 11), in which clear Ni 2*p*_3/2_ multiplet-splitting were observed at ~855.2 and 873.1 eV, indicating the presence of Ni oxides. The significantly larger proportion of Ni 2*p* peaks for Ni^2+^ compared to that for Ni^0^ in the Ni–Fe NP sample can be attributed to the presence of the significantly larger NiO NP, which can be detected relatively easily by XPS compared to the smaller Ni–Fe NP (~5–8 nm) that are well embedded in the carbon fiber substrate. Additionally, the presence of Fe_2_O_3_ in the Ni–Fe NP sample is validated by the deconvolution of the high-resolution Fe 2*p* scan which reveals peaks for both Fe^3+^ and Fe^2+^ (Fig. 2c), giving an Fe^3+/^Fe^2+^ ratio of less than 2, corresponding to the high Fe oxidation state in maghemite (γ-Fe_2_O_3_)^11,44^. It is worth noting that the binding energy of Fe 2*p*_3/2_ in the Ni–Fe NPs is higher than that in both Fe_2_O_3_ and Fe NPs (Supplementary Fig. 12), despite sharing an identical crystal XRD pattern (Fig. 2a), indicating modifications to its electronic structure. The presence of Fe_2_O_3_ was also consistent to the observation of a metal bound O 1*s* peak at 530.6 eV (Fig. 2d). The asymmetric oxidation of the Fe-rich domain in the Janus Ni–Fe NP structure can be ascribed to the oxygen scavenging property and lower electronegativity of Fe (Pauling scale, Fe = 1.83, Ni = 1.91). Additionally, the presence of interface enables partial charge transfer from the Fe-rich domain to Ni-rich domain across the interface due to the electronegativity asymmetry. Hence due to the higher electronegativity of Ni, electron from Fe domain is partially transferred into the Ni-rich domain, protecting the Ni-rich domain from oxidation.

Further investigation of the Ni–Fe NP structure was carried out with X-ray absorption spectroscopy (XAS). Supplementary Figure 13 shows the Ni XANES data collected on the materials compared to key reference materials. The physical mixture of Ni NP and Fe NP (Ni/Fe NP) is a well fit by a combination of 80% metallic nickel and 20% NiO, consistent with previous characterizations (XRD and TEM), indicating the formation of core-shell Ni/NiO NP. On the other hand, the Ni XANES of Ni–Fe NP shift to lower energy consistent with the presence of interface between the γ-Fe_2_O_3_ phase and metallic nickel. This change is not well described by the Ni reference materials (blue arrow) indicating that it has properties that are distinct from any of them. By correlation with the TEM (Fig. 1b) the most plausible explanation is the formation of an interface between Ni and γ-Fe_2_O_3_. The EXAFS data at the Ni edge (Supplementary Fig. 14) are dominated by metallic nickel are substantially dampened probably due to structural disorder. The Fe XAS spectra of the same set of materials are presented in Supplementary Fig. 15. The XANES taken at the Fe edge show a shift to lower energy from the nanoparticle with interface (Ni–Fe NP). This is consistent with the contribution of a reduced Fe phase at the interface between Fe and Ni and is consistent with the XPS. This would be consistent with what was expected from the formation of an interface between metallic nickel and γ-Fe_2_O_3_ interface as observed in the TEM. The EXAFS of these materials are dominated by that of γ-Fe_2_O_3_, which is expected as the majority of the Fe in the sample are in the form of γ-Fe_2_O_3._

### Electrochemical performance

**Fig. 3:**
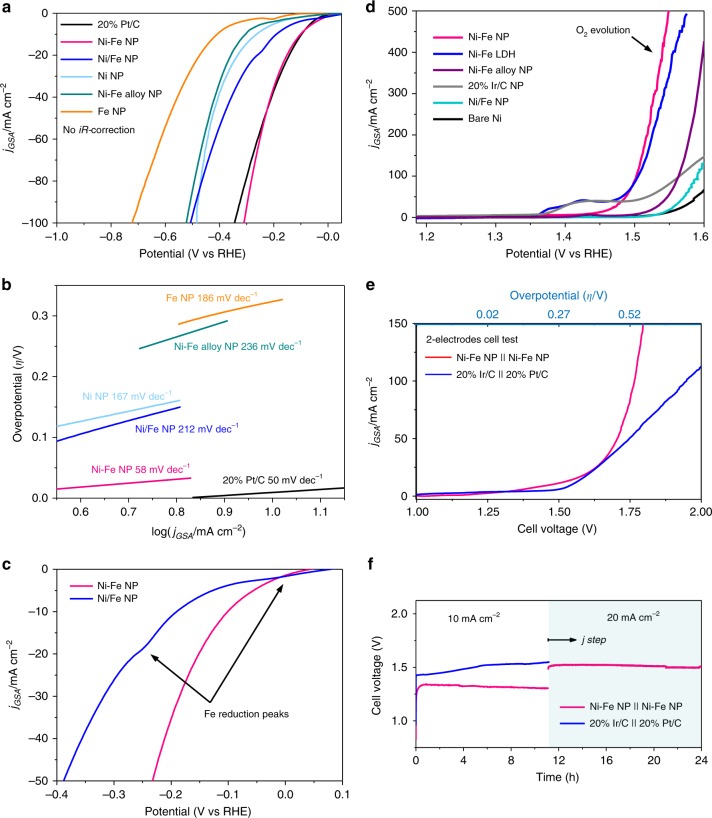
Electrochemistry. **a** HER-LSV curves for Ni–Fe NP, Ni/Fe NP, Ni NP, Ni–Fe alloy NP, Fe NP, and 20% Pt/C electrode (no *iR*-correction). **b** Tafel plots for all nanoparticles and benchmark catalysts. **c** The LSVs shows the presence of metal reduction peaks from Ni/Fe NP electrode. The peaks at 0 and −0.2 V is identical to Fe^3+/2+^ and Fe^2+/1+^ reduction peaks on the HER-LSV of Fe NP electrode shown in **a** and Supplementary Fig. 20. **d** The OER LSV curves for Ni–Fe NP, Ni–Fe LDH, Ni–Fe alloy NP, Ni/Fe NP, 20% Ir/C, and NF electrode. **e** LSV comparing the water-splitting performance of Ni–Fe NP cell and Ir/C-Pt/C cell. **f** The stability test of Ni–Fe NP cell at current of 10 and 20 mA cm^−2^ (magenta trace), the blue trace represent the stability of Ir/C-Pt/C cell. All voltammetry was collected in 1 M KOH, with a scan rate of 5 mV s^−1^.

Figure 3a shows the HER activity of Ni–Fe NP supported on carbon fiber paper (CFP) obtained in 1.0 M KOH by using linear sweep voltammetry (LSV) at a scan rate (*v*) of 5 mV s^−1^. CFP was chosen for HER activity evaluation due to its negligible HER activity. The catalyst mass loading and mole ratio of Ni to Fe were optimized based on the HER performance (Supplementary Figs. 16 and 17), and the best ratio of 5:1 was chosen for HER testing. Shown in Fig. 3a, the Ni–Fe NP exhibits an HER onset potential of −0.18 V vs. RHE (as determined by baseline extrapolation method (Supplementary Fig. 4), accompanied by visual detection of H_2_-gas bubbles formation on the electrode surface. Additionally, the Ni–Fe NPs exhibit a very low HER overpotential (*ɳ*) of 100 mV (without *iR*-corrections) to achieve a current density (*j*) of 10 mA cm^−2^. Only 46 mV is required after the *iR*-correction (Supplementary Fig. 18). This HER performance is comparable to the benchmark Pt catalyst (20% Pt/C) supported on CFP at the same catalyst loading, and outperforms most, if not all, non-precious metal-oxides, -chalcogenides, -nitrides, and -phosphides-based HER catalysts in similar alkaline media (Supplementary Table 2). In contrast, significantly higher HER overpotentials are required for Ni NPs (260 mV) and Fe NPs (410 mV) to achieve 10 mA cm^−2^, suggesting the distinct HER catalytic activity of the Ni–Fe NPs. Consistent trend in performance is also observed in Supplementary Fig. 22a where LSV curves are normalized against the electrochemical active surface area (ECSA) determined from double layer capacitance measurements (Supplementary Figs. 2 and 3). Ni–Fe NP exhibits the lowest *ɳ* of 200 mV for *j*_ECSA_ = −10 mA cm^−2^. The stability of catalyst was also tested under a high current condition (Supplementary Fig. 19a); the test was configured at *j*_HER_ *=* *−*100 mA cm^−2^. Shown by the *E–t* trace (Supplementary Fig. 19b), it is revealed that Ni–Fe NP remains stable at relatively low *η* of 180 mV for an extended period of 10 h at a constant current (−10 mA cm^−2^). Additionally, based on Supplementary Fig. 19d, assuming that all Ni and Fe in the Ni–Fe NP participate in HER catalysis, the turn over frequency (TOF) of Ni–Fe NP at *ɳ* = 200 mV is 0.056 s^−1^ (Supplementary Information for details), an order of magnitude lower than that of Pt (0.9 s^−1^) but comparable to other state-of-the-art metal chalcogenide/phosphide and metal alloy, catalysts such as MoS_2_ (0.02 s^−1^), Ni_5_P_4_ (0.06 s^−1^), and Ni–Mo catalyst (0.05 s^−1^)^45–48^.

Furthermore, the physical stability of Ni–Fe NP was also verified with inductively coupled plasma-mass spectrometry (ICP-MS) measurement of Ni and Fe in the electrolyte (Supplementary Table 3) during a 10 h constant current electrolysis at −50 mA cm^−2^. As shown in Supplementary Table 3, no metal dissolution was observed in the first hour of electrolysis. After 10 h of electrolysis, trace amount of Fe (70 μg L^−1^) was detected in the electrolyte which corresponds to only 1.5 wt% of Fe in the fresh electrode. Additionally, Supplementary Fig. 19d shows the loss of HER activity is minimal, which is validated by the LSVs obtained before and after the stability testing. The stability is also confirmed by HAADF-STEM (Supplementary Fig. 19c), showing insignificant changes in the physical features of Ni–Fe NP. The crystal structure of the Ni–Fe NPs is also preserved after the stability test, as confirmed by XRD (Supplementary Fig. 27a).

To investigate the HER reaction kinetics, Tafel plots were derived from the LSVs and shown in Fig. 3b. The Ni–Fe NP exhibits a much smaller slope of 58 mV dec^−1^ than Ni NP (167 mV dec^−1^), Fe NP (186 mV dec^−1^), and Ni/Fe NP (212 mV dec^−1^), and an extremely large exchange current density of 1.58 × 10^−3^ A cm^−2^ which even matches with Pt/C catalyst in acidic media^49,50^. This slope value is also distinctly different from the previously reported Ni–Fe-based catalysts, which typically exhibit Tafel slopes above 80 mV dec^−1^ ^51,52^ and approaches 20% Pt/C-CFP (Tafel slope: 50 mV dec^−1^), suggesting the HER at the Ni–Fe NP follows a Volmer–Heyrovsky HER mechanism^50^.

To understand the role of the interface between Ni and γ-Fe_2_O_3_ for HER_,_ a physical mixture of Ni NPs and Fe NPs (denoted as Ni/Fe NPs) and an Ni–Fe alloy mixture (Supplementary Fig. 9, denoted as Ni–Fe alloy NP) were prepared at a molar ratio of 5 to 1, and their HER activities were evaluated. The physically mixed Ni/Fe NP was produced by drop-casting Ni(oleate)_2_ and Fe(oleate)_2_ at different stages, instead of the standard pre-mixing procedure of Ni^2+^ and Fe^2+^ with the oleate anion prior to the micellization step (Supplementary Fig. 1). The Ni–Fe alloy NP were prepared by annealing the precursor of Ni–Fe NP in high temperature H_2_ atmosphere to achieve better alloying. Shown in Fig. 3c, the Ni/Fe NP and Ni–Fe alloy NP require overpotentials of 112 and 307 mV, respectively, to achieve 10 mA cm^−2^, which are significantly higher than that required for Ni–Fe NP. Importantly, it is noted that the Fe^3+^ reduction peaks at 0 and −0.2 V observed in both Ni/Fe NPs (Fig. 3c) and Fe NPs (Supplementary Fig. 20) are not present in the LSV of Ni–Fe NPs, suggesting the redox behavior of the interconnected γ-Fe_2_O_3_ is altered as a result of the formation of Ni-γ-Fe_2_O_3_ interface.

Intriguingly, the Ni–Fe NP also exhibits extraordinary activity towards OER. We further examined the OER performance of the Ni–Fe NPs supported on nickel foam substrate (NF, Fig. 3d) and CFP (Supplementary Fig. 21). Nickel foam was used as substrate due to its excellent electrochemical properties, e.g. corrosion resistance against OER, electrical conductivity, 3-D porous structure, and robust mechanical strength^11,22,52^. The state-of-the-art NiFe layered double hydroxide (NiFe-LDH) electrocatalysts were also synthesized according to the established method to provide comparison^14^. Figure 3d shows that OER process catalyzed by Ni–Fe NP reaches a current density of 10 mA cm^−2^ at a low overpotential of 210 mV. Higher current densities of 20 mA and 100 mA cm^−2^ were achieved at *η* of 230 and 270 mV, respectively. These values outperform 20% Ir/C (Fig. 3d) as well as most reported transition metal oxides, chalcogenides, phosphides, and nitrides electrocatalysts (Supplementary Table 4). The TOF at *η* of 350 mV, by assuming all Ni and Fe in Ni–Fe NP participate in catalysis, is calculated to be 0.052 s^−1^ (Supplementary Information for details), which is comparable to benchmark 20% Ir/C (0.027 s^−1^) and electrodeposited NiFe/NF (0.075 s^−1^) at *η* of 400 mV, respectively^11^. It is worth noting that due to the small proportion of Ni–Fe interfaces on the Ni–Fe NP surface, the amount of Ni–O–Fe active sites are far less than the loaded amount of Ni and Fe, and therefore the actual TOF should be higher than the calculated values. Additionally, the LSVs were also normalized against ECSA (Supplementary Fig. 22b). Consistent with Fig. 3d, Supplementary Fig. 22b shows that Ni–Fe NP exhibits the highest OER performance with *j*_ECSA_ = 10 mA cm^−2^ achieved at *η* of 300 mV, which is also higher than electrodeposited NiFe/NF^11^. These comparisons indicate the crucial role of Ni-γ-Fe_2_O_3_ interface present in the Ni–Fe NP for OER electrocatalysis.

Supplementary Figure 23 shows that the Tafel slope of Ni–Fe NP is lower than that of NiFe-LDH and the linearity of the plot is also maintained at high *j*, indicating fast electron transfer and mass transport properties of the catalyst^12,53^. Supplementary Figure 24 shows chronopotentiometric response of Ni–Fe NP for OER with *j* increasing gradually from 50 mA to 500 mA cm^−2^ in nine-equal current steps of 50 mA cm^−2^. The potential response was observed to level off quickly on each stepping with high consistency, suggesting excellent mass transport properties as well as physical stability^11^. Excellent stability of the Ni–Fe NP for prolonged OER is illustrated in Supplementary Fig. 25a, b. Further, ICP-MS, HAADF-STEM, LSVs, and XRD analyses of Ni–Fe NP following OER stability tests also show negligible dissolution of the catalyst during OER (Supplementary Table 5), and no significant change to structure and OER activity (Supplementary Figs. 25c–d and 27b), compared to fresh Ni–Fe NP electrode.

Importantly, Fig. 3d shows the Ni oxidation peak prior to the onset of OER is greatly suppressed in the Ni–Fe NP configuration (Supplementary Fig. 26 for greater magnification of the Ni peak). The oxidation peak corresponds to the oxidation of Ni from low valence states (Ni^0^, Ni^2+^) to high valence states (Ni^3+^ or Ni^4+^), the latter are believed to be the active sites for OER^54,55^. The greatly reduced Ni oxidation peak, compared to that observed for Ni NP, Ni/Fe NP, and Ni–Fe alloy NPs (Fig. 3d), suggests that OER active sites already exist in the Ni–Fe NP, rather in operando formed. Compared to the structures and electrochemical results obtained for Ni NP, Ni/Fe NP, and Ni–Fe alloy NPs, where no Ni/Fe_2_O_3_ interface present, it is clear that the Ni-γ-Fe_2_O_3_ interface play a major role as the OER active site. Similar behavior is observed for HER, where the Fe reduction peaks prior to the onset of HER currents are observed for Ni/Fe NP (Fig. 3c) and Fe NP (Supplementary Fig. 20) but not for Ni–Fe NP, suggesting that the HER active sites in Ni–Fe NP are also present at the start of HER. Collectively, these results suggest that both OER and HER occur at the similar sites at the interface of Ni–Fe NP. It is also important to note that the presence of metallic Ni enables fast electron transport within the nanoparticle that is required for efficient electrocatalysis.

Further, Ni–Fe NP was used as both the anode and cathode in a whole cell water electrolyzer (details in Methods). The polarization curve of the Ni–Fe NP cell is recorded and compared to the cell constructed using benchmark noble metal catalysts, 20% Ir/C (OER) and 20% Pt/C (HER). Shown in Fig. 3e, the Ni–Fe NP cell exhibits superior performance to the Ir/C || Pt/C cell. The cell potential required for Ni–Fe NP cell to achieve 10 mA cm^−2^ is only 1.47 V (1.55 V without *iR*-correction; Supplementary Fig. 28), which is among the lowest bi-functional water-splitting catalysts reported recently (Supplementary Table 6). Based on the calculated HER and OER Faradaic efficiency of approximately ~100% at *j* = 10 mA cm^−2^ (Supplementary Fig. 31), the energy efficiency of the Ni–Fe NP cell is calculated to be 83.7% with *iR-*correction (79.4% without *iR-* correction). The Ni–Fe NP cell also shows excellent stability during the 24 h bulk water electrolysis at *j* = 10 and 20 mA cm^−2^ (Fig. 3f). In comparison, the Ir/C || Pt/C cell requires a cell voltage of 1.62 V to achieve 10 mA cm^−2^ and the performance was observed to deteriorate after 2 h, which is consistent to previous report^4^. In addition, to further demonstrate the Ni–Fe NP electrochemical stability against power interruptions, an accelerated degradation test (ADT) was performed by alternating the polarity of the electrode repeatedly. In this experiment, the electrolysis current is switched between 100 mA cm^−2^ (OER) and −100 mA cm^−2^ (HER), and the Ni–Fe NP electrode is subjected to anodic oxidation for OER for 600 s before switching to HER, and vice versa. Supplementary Figure 29 displays the stable potential response throughout the 4200 s duration of the ADT, confirming the exceptional stability of Ni–Fe NP as a reversible, bi-functional catalyst for water electrolysis under intermittent conditions.

### DFT simulation

**Fig. 4:**
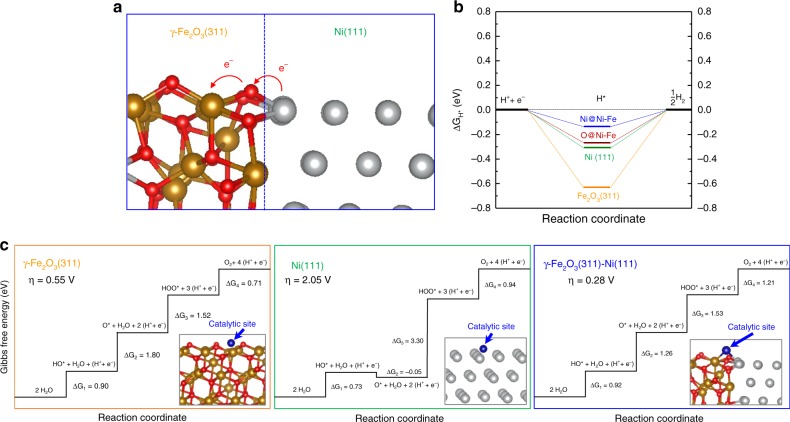
Theoretical understanding. **a** Optimized interface structure of the Ni–Fe heterojunction. **b** Standard free energy diagram of the HER process on the γ-Fe_2_O_3_ (311) and Ni(111) surfaces and their interface in the Ni–Fe heterojunction. **c** Standard free energy diagram of the OER process on the γ-Fe_2_O_3_ (311) and Ni(111) surfaces and their interface in the Ni–Fe heterojunction. The insets show the optimized structures and the catalytic sites for OERs. Key—brown: iron, red: oxygen, gray: nickel, and blue: catalytic site.

To offer insights into the superior HER and OER performance of the Ni–Fe NP, the first-principles DFT calculations were conducted (more details in Supplementary Information). The interface model between the γ-Fe_2_O_3_ (311) and Ni (111) surfaces (Fig. 4a) was built based on the experimentally observed STEM-HAADF image of a single Ni–Fe NP (Fig. 1b). The Fe and Ni atoms are coupled via the bridge O atoms at the interface as highlighted in Supplementary Fig. 6. The strong interaction between Ni and O at the interface can be further confirmed by their overlapped O 2*p* and Ni 3*d* states around the Fermi energy level (Supplementary Fig. 31).

The Δ*G*_H*_ is a key descriptor for evaluating the performance of HER electrocatalysts^56^. The DFT results reveal that the H atom can either adsorb on the top site of O atoms in γ-Fe_2_O_3_ (311) or the *fcc* site of Ni(111) with the Δ*G*_H*_ of −0.62 and −0.31 eV, respectively. It is worth noting that our Δ*G*_H*_ value on the Ni(111) surface is almost identical to that calculated by Nørskov et al.^56^. The negative Δ*G*_H*_ values suggest that the activity of both surfaces are too high for HER. At the interface of the Ni–Fe NP, however, both the interfacial O and Ni atoms have more optimal activity to the HER as demonstrated by corresponding Δ*G*_H*_ of −0.27 and −0.14 eV, respectively (Fig. 4b). It suggests that both the activities of the interfacial Ni and O atoms are reduced due to the formation of Ni–O bond and thereafter the charge transfer, which is beneficial to the HERs, as observed in experiments (Fig. 3a).

To understand the OER activities, the change of the Gibbs free energies, Δ*G*_*n*_ (*n* = 1, 4), for the four fundamental OER steps were calculated since the magnitude of *ɳ* has been demonstrated to be the difference of the practical potential (maximum Δ*G*_*n*_ over the charge *e*) and the standard Nernstian potential (1.23 V vs. SHE). Thus, the Δ*G*_*n*_ values of intermediates need to reach the minimal difference between each other to reduce the *ɳ*. The calculated Δ*G*_*n*_ on the γ-Fe_2_O_3_(311), Ni(111), and heterojunction are shown in Fig. 4b–d. The theoretical results indicate that the γ-Fe_2_O_3_ itself is a good OER electrocatalysts with the theoretical *ɳ* of 0.55 eV. The rate-determination step is the formation of O^*^, in which the binding strength of Fe and O^*^ is considerably weak. As a comparison, the Ni metal possess large *ɳ* of 2.05 V with the rate-determination step for the formation of OOH^*^ intermediate. This is largely due to much stronger binding between the surface Ni atom and the O^*^ intermediate (Supplementary Table 8). At the interface of Ni–Fe NPs, the binding energy of O^*^ is optimized since it adsorbs at the bridge site between Fe and Ni. As such, the smallest theoretical *ɳ* of 0.28 V is attained among three systems, which greatly matches the experimental observation (Fig. 3d). Our theoretical studies, therefore, demonstrate that only the interface of the Ni–Fe NPs has the superior OER performance (Supplementary Fig. 32).

## Discussions

The unusually high electrocatalytic HER activity of the Ni–Fe NP can be ascribed to the electronic coupling effect arising from the interface due to the asymmetrical distribution of Ni and Fe_2_O_3_ in the nanoparticle. Based on the Bader charge analysis result (Supplementary Table 7), Ni atoms at the interface are slightly oxidized due to the direct interaction with O anions (Fig. 4a). The Δ*G*_H*_ of −0.30 eV on the Ni(111) indicates that the surface Ni atoms bind H* too strongly. Consequently, relatively large overpotential is required for the formation and desorption of H_2_ product. The slight oxidation of surface Ni atoms at the interface can reduce their activity, which can optimize the Δ*G*_H*_ value to approach the optimal value of 0.0 eV. Moreover, the presence of metallic Ni provides metallic-type electron conduction which warrants facile electron transport towards the active HER sites at the interface as revealed by both experimental and computational results. Concomitantly, the HER onset of Ni–Fe NP is markedly lowered to RHE potential comparable to that of Pt/C due to the significantly lowered energy barrier for the initial Volmer step^57^ that is determined by electron transfer kinetic (Eq. 1):

1 $${\mathrm{H}}_2{\mathrm{O}} + {\mathrm{e}}^- \to {\mathrm{H}}^ \ast + {\mathrm{OH}}^- \left( {\mathrm{{aq}}} \right).$$

The following H_2_ evolution was then promoted by the unique Ni–O–Fe configuration at the interface (Fig. 4a). Experimentally, this is manifested in distinctly different Tafel slope in the order of 20% Pt/C ~Ni–Fe NP ≪ Ni NP < Fe NP, suggesting a significantly altered secondary H_2_ evolving step, in contrast to Ni NP and Fe NP.

Intriguingly, the synergy between Ni and Fe_2_O_3_ at the interface of their NPs can also benefit the OER process. Based on the analyses of Gibbs free energy diagrams, the Fe^3+^ cation and Ni^0^ atom possess too weak or too strong activities to the OER, respectively. The spontaneous interaction of the OER intermediates formed at the bridge site between Ni and Fe can greatly optimize their Gibbs free energies, improving the OER performance as evidenced by the theoretically optimized atomic structures shown in Fig. 4c. Moreover, the unique Ni–O–Fe configuration at the interface as indicated in Fig. 4a provides a great platform to achieve multi-site functionality for the electrocatalysts. The multi-site functionalization can offer several active sites to stabilize one adsorbed state or transition state without stabilizing others, which can, therefore, break the limit of energy scaling relations of OER to lower the overpotential^58^. Previous studies reveals that the Δ*G*_*2*_ + Δ*G*_*3*_ values are within 3.2 ± 0.2 eV range due to their scaling relation among reactive intermediates^38^. Our Δ*G*_*2*_ + Δ*G*_*3*_ values on the γ-Fe_2_O_3_(311) and Ni(111) surfaces are 3.32 and 3.25 eV, respectively, which are the examples of this scaling relations. At the interface of Ni–Fe NPs, the OOH* intermediate is stabilized through the formation of hydrogen bonding between its H atom and neighboring interfacial O atom (Supplementary Fig. 33). Consequently, the energy scaling relations are circumvented with a reduced Δ*G*_*2*_ + Δ*G*_*3*_ value of 2.79 eV and smallest theoretical *ɳ* of 0.28 V. This multi-site functionalization mechanism found in our system can also explain other Ni–Fe-based OER electrocatalysts with the overpotential lower than the theoretical limit (~0.3 V) due to the scaling relation, such as LDH, metal organic framework, or amorphous mixture^11,14,57,59^.

In summary, this work demonstrates that the introduction of asymmetry in an electrocatalyst structure could induce unprecedented synergistic effect for electrocatalysis. Through this approach, we have overcome the practical limitation of Ni–Fe mixed oxides for overall water electrolysis due to the poor HER activity. Additionally, having similar active sites for both OER and HER results in the preservation of catalyst structure and activity against electrode corrosion induced by power interruptions, which is ideal for a water electrolyzer powered by intermittent renewable energy sources. Beyond, it is also our hope that this multi-site functionality catalyst design can help to expedite the conception-to-commercialization process of other multi-metallic nanoparticle electrocatalysts with different compositions and structures that exhibit distinct interfaces for various electrolytic applications such as CO_2_ reduction reactions and nitrogen reduction reactions.

## Methods

### Nanoparticles and electrodes preparation

To prepare Ni–Fe NP, initially 5 mmol of Ni(NO_3_)_2_.6H_2_O (UNIVAR grade, Ajax Finechem, Australia) and 1 mmol Fe(Cl)_2_.4H_2_O (Sigma-Aldrich, USA) were dissolved into a mixture of 2 mL of de-ionized water and 1 mL of ethanol. Four grams of Na(oleate) (TCI, Japan) was dissolved into a separate mixture of 4 mL de-ionized water and 3 mL of ethanol. The two mixtures were then mixed in a round-bottomed flask to yield thick and waxy green-yellow substances, and into the mixture, 14 mL of hexane was added. Upon the addition of hexane, immediate transfer of the colored metallic elements into the hexane phase was observed (Supplementary Fig. 1); the mixture was then stirred at 400 r.p.m. for 60 min. Two well-separated layers of water (colorless/opaque) and colored hexane layer (dark-green) were obtained at the end of stirring. Other oleate complexes with different Ni and Fe ratios were made by the same method by adjusting the mole ratio between Ni and Fe salts precursors to yield metal-oleate complex solution with a concentration of 0.43 mmol_(metal)_ mL^−1^ (6 mmol of metals in 14 mL of hexane).

Electrodes were prepared by employing substrate materials such as CFP (FuelCell Store, Spectracarb 2050A-1535, USA) and nickel foam (NF, thickness: 1.6 mm, bulk density: 0.45 g cm^−3^, Goodfellow, UK). The substrates were modified with metal-oleate complexes dissolved hexane solution via the drop-casting method. For the preparation of Ni NP, Fe NP, and Ni–Fe NP on CFP, 180 μL of the corresponding metal/metals oleate complex in hexane was drop casted onto 3 cm^2^ of CFP. In case of NF, 300 μL (7.5 mg of metal) were drop casted onto 0.22 g of NF (GSA = 3 cm^2^, Supplementary Information for conversion calculation). For the preparation of Ni/Fe NP onto CFP electrode, 150 μL of Ni(oleate)_2_ was drop casted onto a 3 cm^2^ CFP, followed by 30 μL of Fe(oleate)_2_. The substrate was then dried under bench-top condition until the remaining hexane was evaporated and a waxy film was formed at the electrode surface. The modified substrates were subsequently annealed in Ar-protected horizontal tube furnace at 350 °C for 2 h at a ramp rate of 10 °C min^−1^ with an Ar-flow rate of 5 mL min^−1^. Upon completion, the annealing chamber was allowed to cool down naturally to room temperature. For the preparation of Ni–Fe alloy NP, following the micellization step (Supplementary Fig. 1), the Ni–Fe oleate complex were further incubated at 70 °C for 7 h to allow further equilibration of Ni^2+^ and Fe^2+^ distribution within the micellar metal complex^8^, then following immobilization step the modified substrates were annealed under in H_2_ atmosphere at 700 °C for 2 h at a ramp rate of 10 °C min^−1^ with a gas flow rate of 5 mL min^−1^. The annealed electrodes were washed ultrasonically with Milli-Q water to remove the loosely bound nanoparticles for 5 min. Catalyst mass loadings (*m*_l_) of CFP and NF modified with metal nanoparticles were 1.5 and 2.5 mg cm^−2^, respectively, and these values were determined according to the calculations shown in the Supplementary Information, unless otherwise stated. To verify the mass loading nanoparticles on CFP, thermogravimetric (TGA) measurement was carried out on 0.5 cm × 0.5 cm of Ni–Fe NP/CFP and the result is shown in Supplementary Fig. 30. TGA measurement was carried out with a temperature ramp rate of 10 °C min^−1^ with a measurement range of 100–1000 °C in air to remove all of the non-metal components of the electrode. It is shown that at the end of the measurement 0.41 mg of residual weight was observed, corresponding to *m*_l_ of 1.64 mg cm^−2^. The slight discrepancy can be attributed to the weight gains from the formation of metal oxides.

The noble metal modified electrodes were prepared through drop-casting method. An ink of 20% Pt/C was prepared by the addition 10 mg of 20% Pt/C or 20% Ir/C (Premetek, USA) into 1.96 mL of 50% ethanol solution containing 0.04 mL 5% Nafion binder solution (Sigma-Aldrich, USA). The ink was then loaded onto substrate and dried under vacuum at 40 °C, resulting in catalyst mass loading of 3.0 mg cm^−2^.

### Electrochemical characterization

All electrochemical measurements were carried out with a CHI 760 electrochemical workstation (CH Instruments, USA). Electrochemical measurements were performed with a standard three-electrode cell configuration composed of working electrode, graphite rod counter electrode, and standard calomel electrode (SCE) as the reference electrode, unless otherwise stated. The potential of the SCE reference electrodes were routinely calibrated before and after experiments against a SCE that is conserved in saturated KCl solution. For all electrochemical measurement, the recorded SCE potential was converted into RHE using the following formula, *E*_RHE_ = *E*_SCE_ + 0.241 + 0.059 × pH. OER and HER polarization curves were recorded at the scan rate of 5 mV s^−1^, in 1 M KOH at 25 °C with 95% *iR-*corrections using the automated *iR*-correction function of the potentiostat^60^. Two-electrode water electrolysis cell was constructed from two NF electrodes modified with Ni–Fe NP with mass loading of 2.5 mg cm^−2^ in 1 M KOH. Energy efficiency of the cell was calculated using the following equation:

$$\eta _{{\mathrm{electrolyzer}}} = {E}_{{\rm{f}},{\rm{o}}}/{V}_{{\rm{e}},{\rm{i}}},$$

where *E*_f,o_ = 1.23 V; *V*_e,i_ is the input voltage required to drive the electrolysis at the current density of interest. The energy efficiency calculated in this study was obtained at *j* = 10 mA cm^−2^.

## Supplementary information

Supplementary Information

## Data Availability

The data that support the plots within this paper and other findings of this study are available from the corresponding author on request.
