# Supplementary material for: Overall electrochemical splitting of water at the heterogeneous interface of nickel and iron oxide
Source: Nat Commun. 2019 Dec 6;10:5599. doi: 10.1038/s41467-019-13415-8 (PMC6898202; doi:10.1038/s41467-019-13415-8)
Supplement: Supplementary file 1 — Supplementary Information [file 41467_2019_13415_MOESM1_ESM.pdf]

Supplementary Information

For

Overall Electrochemical Splitting of Water at the Heterogeneous Interface of Nickel and Iron Oxide

Suryanto et al.

Supplementary Figures

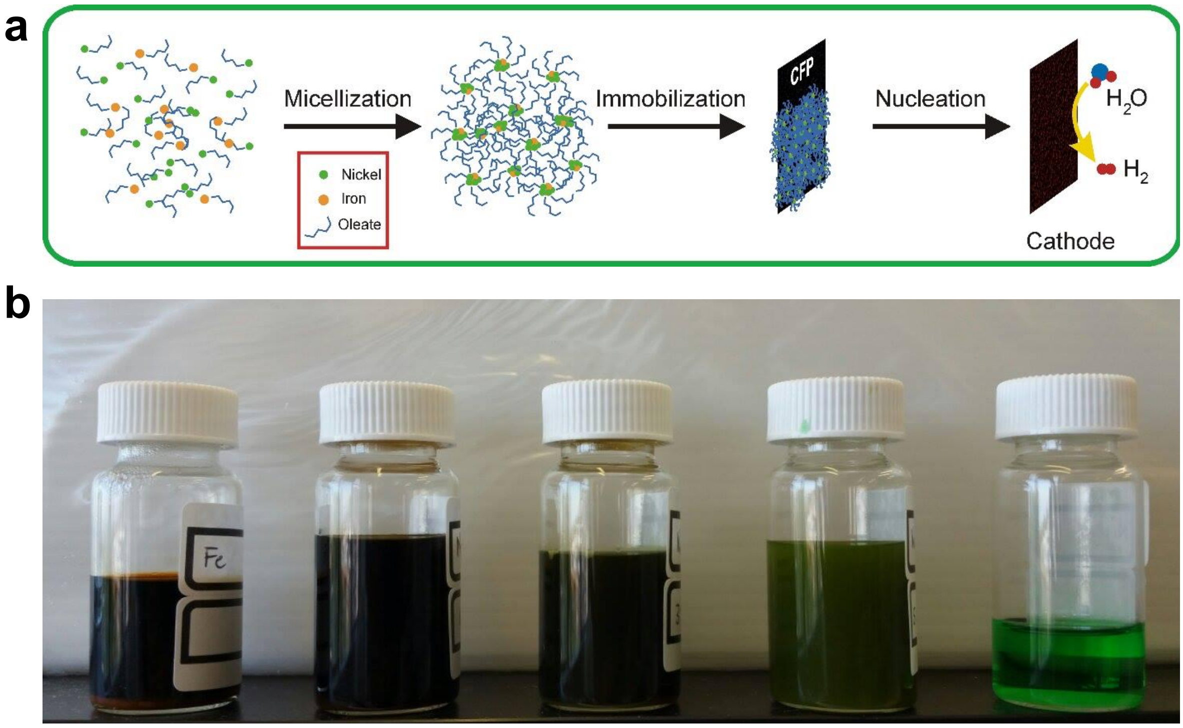

Supplementary Figure 1. The general synthetic procedure of supported nanoparticles

(a) All of nanoparticles were synthesized *via* sodium oleate mediate nano-vesicles (micelles) formation containing metal ions. The formed micelles were immobilized on conductive substrates (CFP or NF) and annealed at 350°C under Ar-gas protection to induce thermal reduction of metal ions into metal nanoparticles. (b) Micelle stabilized metal ions in hexane, from left to right: Fe, Ni_1Fe, Ni_3Fe_1, Ni_5Fe_1 (Ni-Fe NP) and Ni.

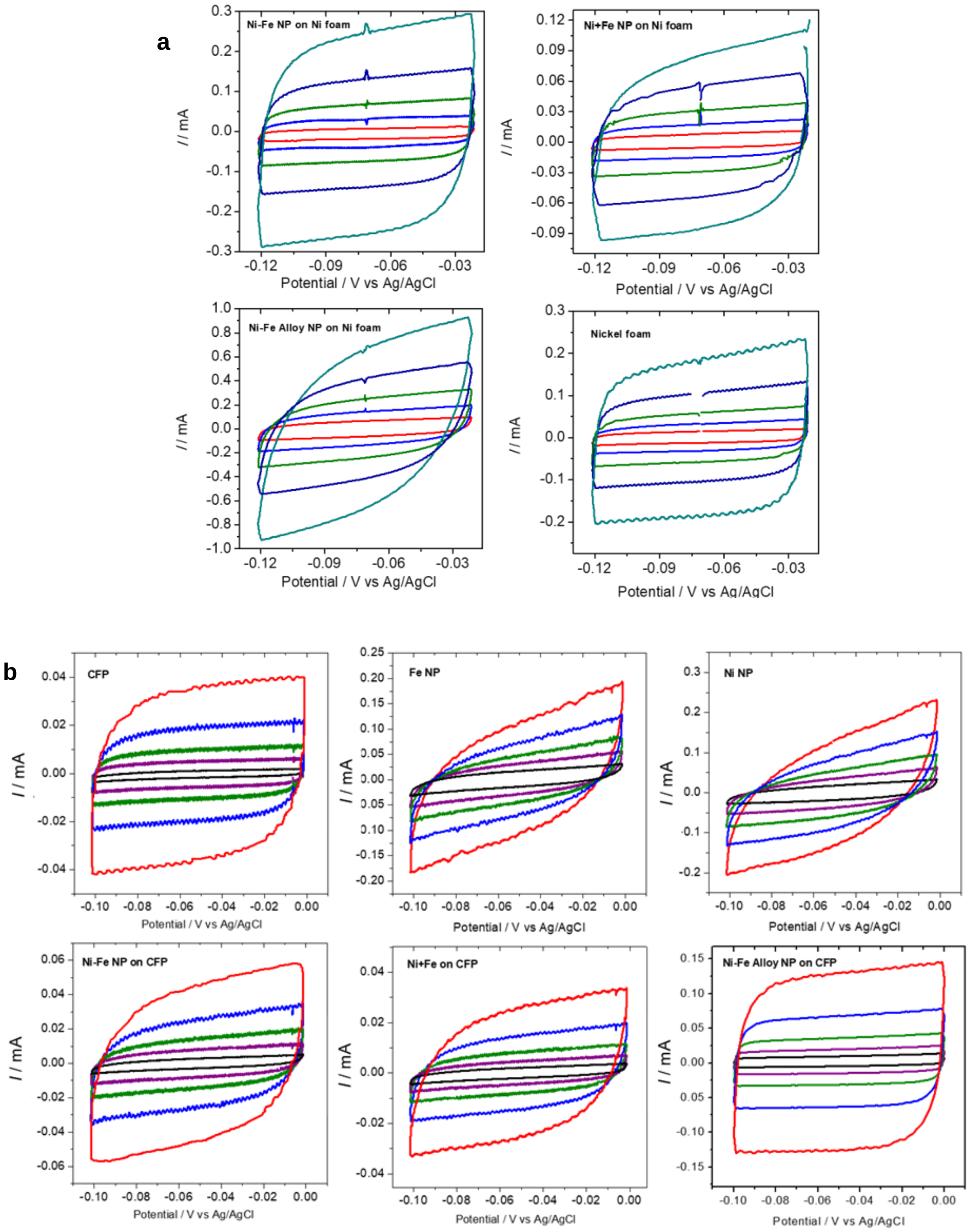

Supplementary Figure 2. Double layer capacitance measurement in 1 M KOH to determine electrochemically active surface area (ECSA)

(a) NF supported catalysts and (b) CFP supported catalysts. The voltammograms were measured in non-Faradaic region and a series of scan rate ranging from 0.01 V s^{-1} to 0.2 V s^{-1} were employed.

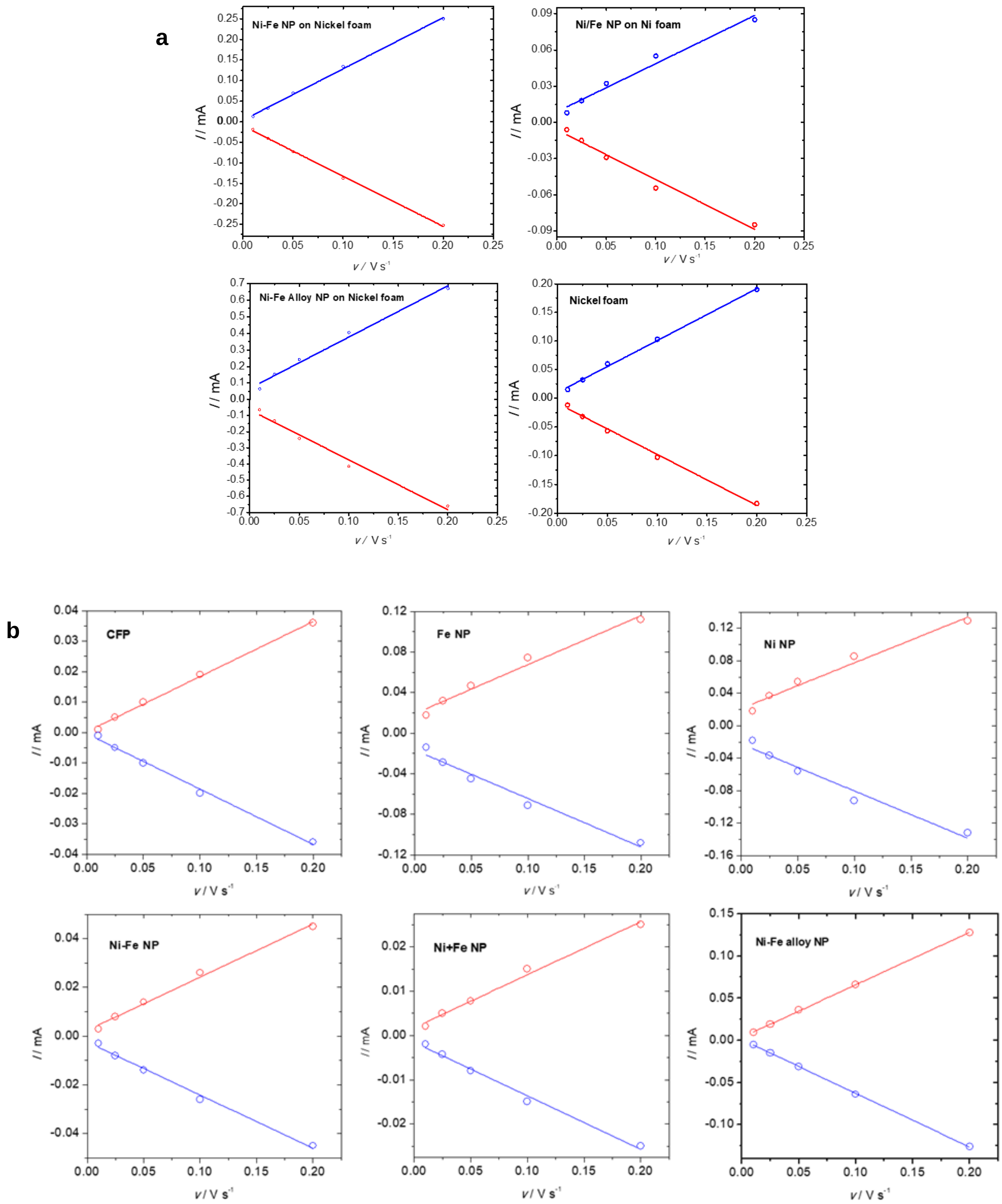

Supplementary Figure 3. The cathodic and anodic charging current plots

(a) The cathodic and anodic charging current plots obtained at $E = -0.07$ V vs Ag/AgCl in Supplementary Figure 2a as a function of scan rate. (b) (a) The cathodic and anodic charging current plots obtained at $E = -0.05$ V vs Ag/AgCl in Supplementary Figure 2b as a function of scan rate.

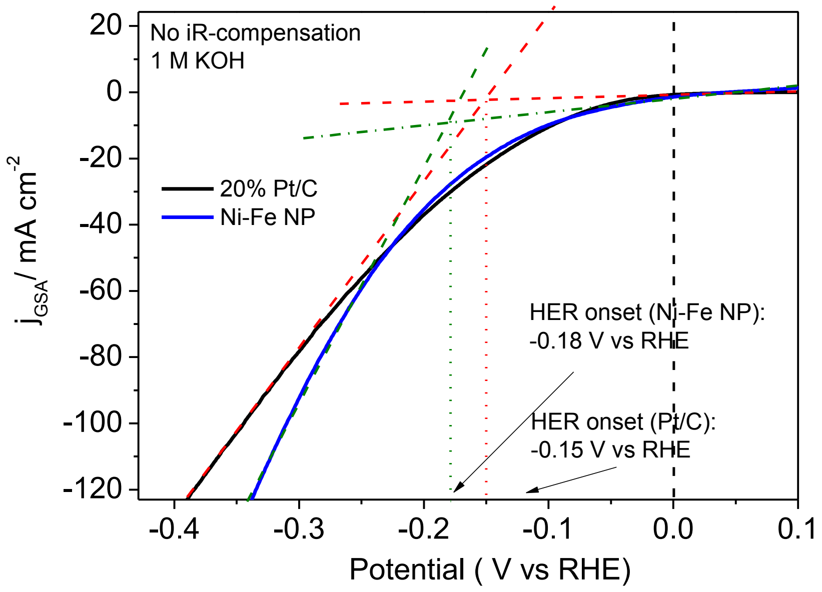

Supplementary Figure 4. Method for determination of onset potential.

Shown above is the updated approach to define onset potentials within the manuscript. This approach is now also used to determine OER onset potentials.

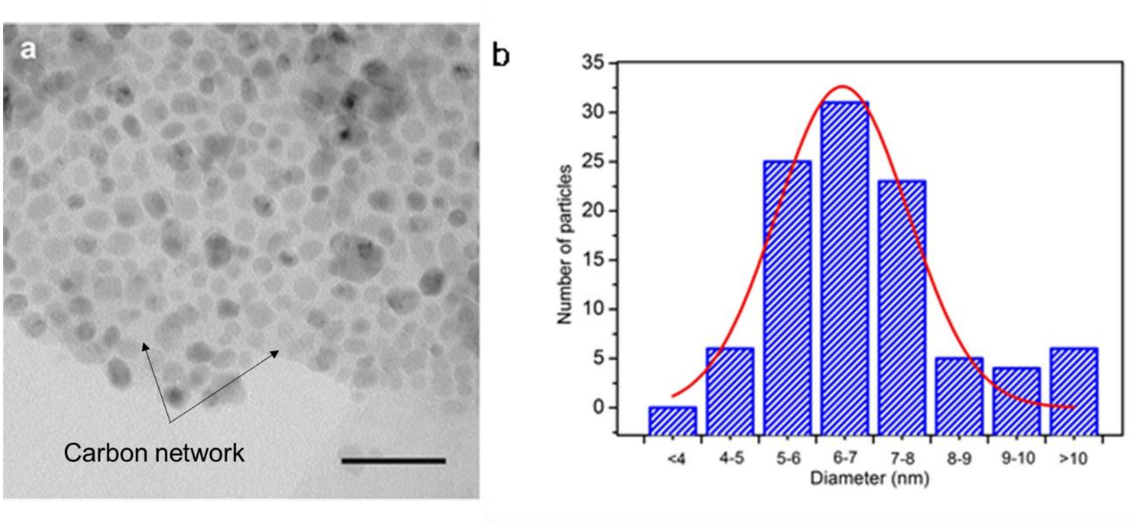

Supplementary Figure 5. TEM characterizations and determination of particle size distribution.

(a) TEM image of typical nanoparticles in Ni-Fe NPs samples (50 nm – scale bar); (b) The size distribution histogram showing the average size of 100 Ni-Fe NPs.

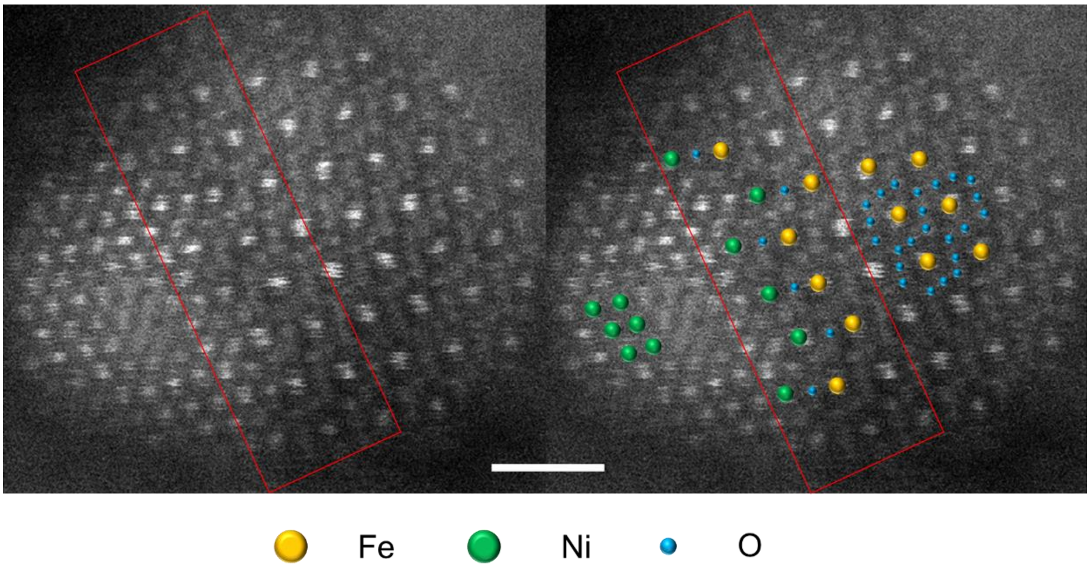

Supplementary Figure 6. STEM-HAADF images

Image of the single nanoparticle without (left) and with (right) Ni, Fe and O superimposed points for comparison. The red box highlights the interface. Scale bar = 1 nm).

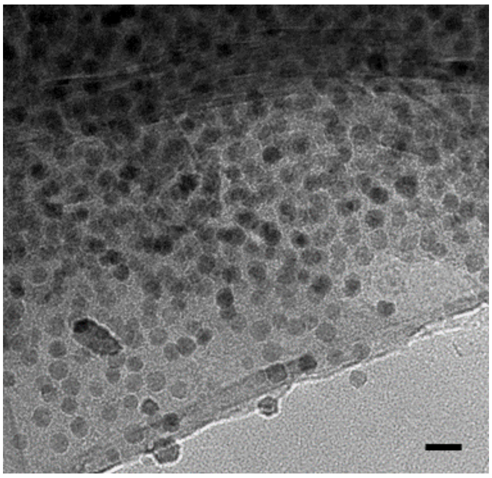

Supplementary Figure 7. TEM microscopy of Fe nanoparticles

TEM images of the as synthesized Fe nanoparticles (Fe NP) supported on CFP. The images shows that the average size of the Fe NP are around 10 nm (scale bar: 20 nm).

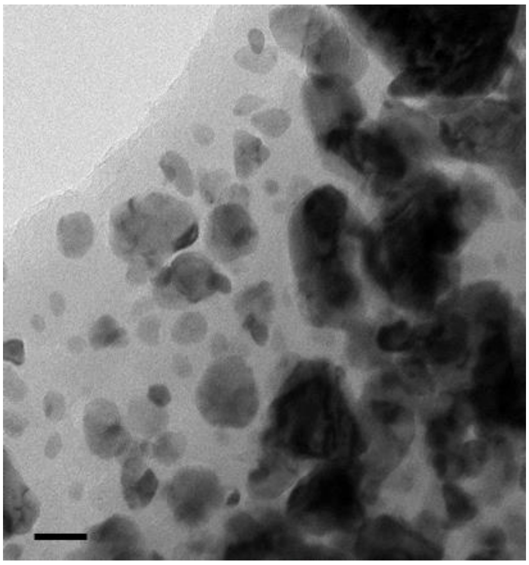

Supplementary Figure 8. TEM microscopy of Ni nanoparticles

TEM images of the as synthesized Ni nanoparticles (Ni NP) supported on CFP. The images shows that the average size of the Ni particles are more than 50 nm (scale bar: 50 nm). The larger particle are caused by the formation of nickel oxides.

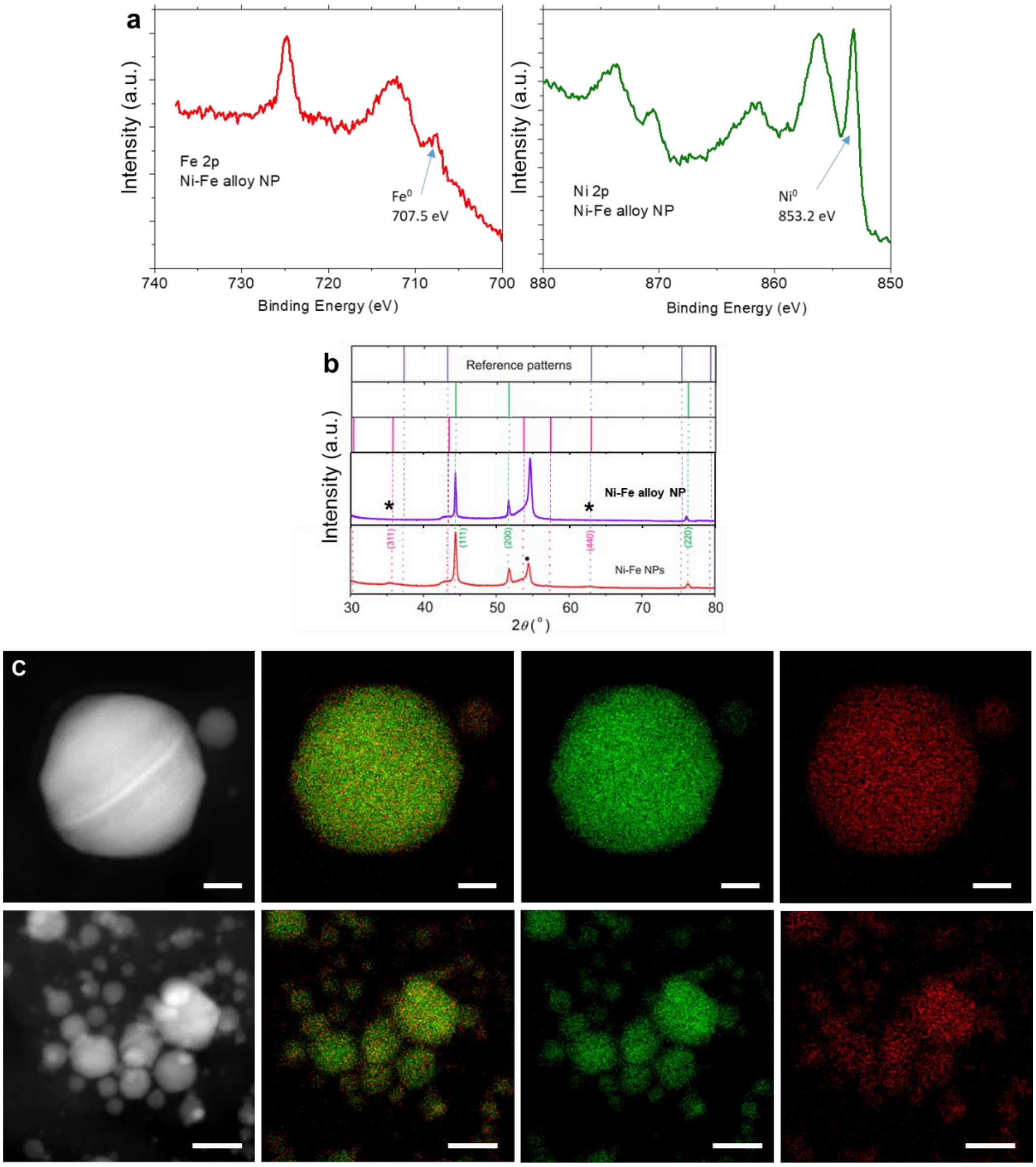

Supplementary Figure 9. Physical characterizations of the synthesized Ni-Fe alloy nanoparticles

(a) High resolution Fe 2p (red) and Ni 2p (green) X-ray photoelectron spectroscopy, indicating the formation of metallic (Fe^0 and Ni^0) nanoparticles; (b) X-ray diffraction spectra (XRD), showing the absence of γ-Fe_2O_3 as indicated by (*) in the spectra; (c) STEM-EDS imaging of the synthesized Ni-Fe NP (green color indicate Ni and red color indicate Fe; scale bars for the single particle image: 20 nm, scale bares for large scale imaging: 50 nm), the homogeneous distribution of Ni and Fe within the particle indicates the successful alloying of Ni and Fe.

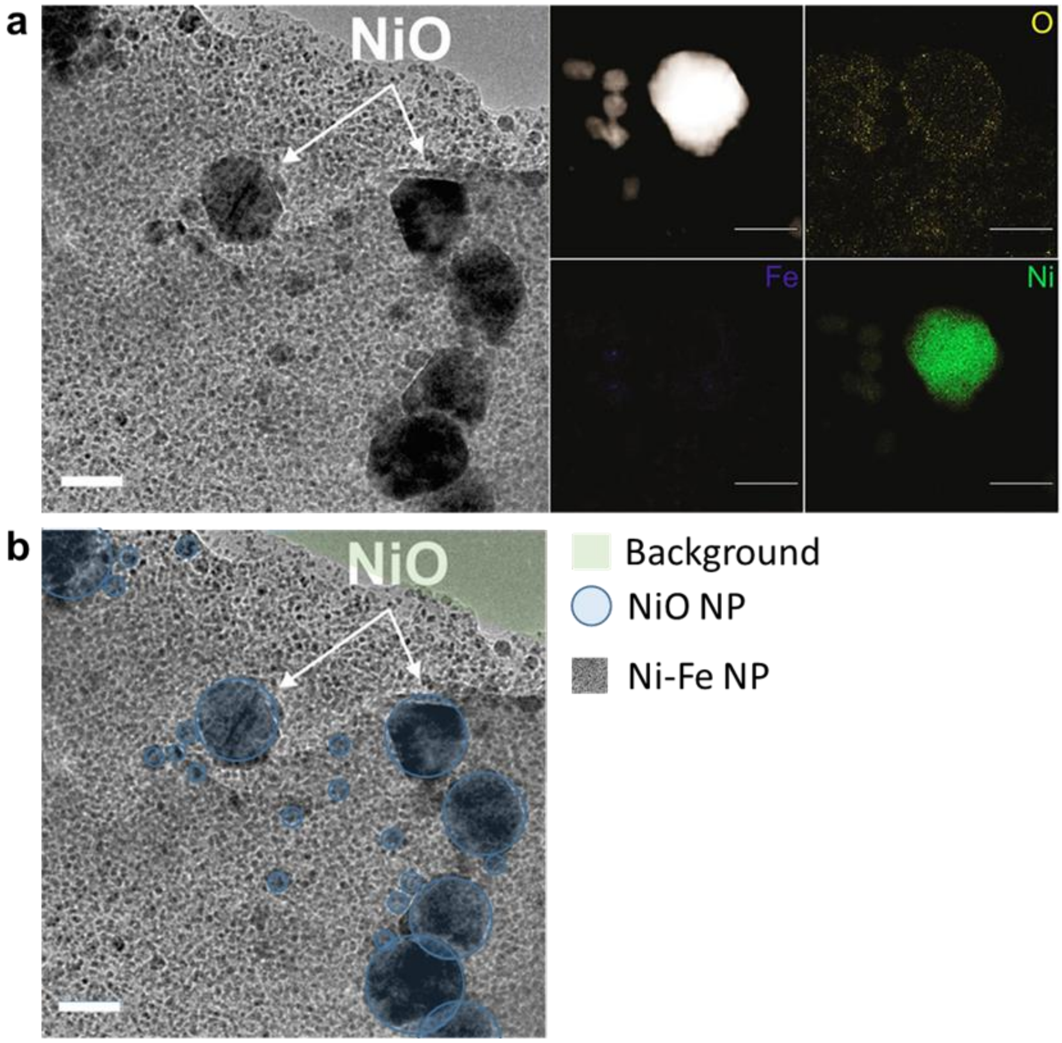

Supplementary Figure 10. STEM – EDS analysis of Ni-Fe NP

(a) Showing the unintended formation of core-shell Ni/NiO NP in Ni-Fe NP detected by HR-TEM (left) and EDS (right), (scale bars: 50 nm). The EDS revealed that Fe was not detected in the core-shell Ni/NiO NP; (b) The percentage of NiO NP was analysed based on % area coverage of NiO NP from the total area of 150,700 nm^2 in this low magnification micrograph: Area% NiO NP = Area (NiO NP) / Area (Sampling area – Background)×100%. Based on areal calculation (shown above), there are ~ 17% of NiO NP in the Ni-Fe NP sample.

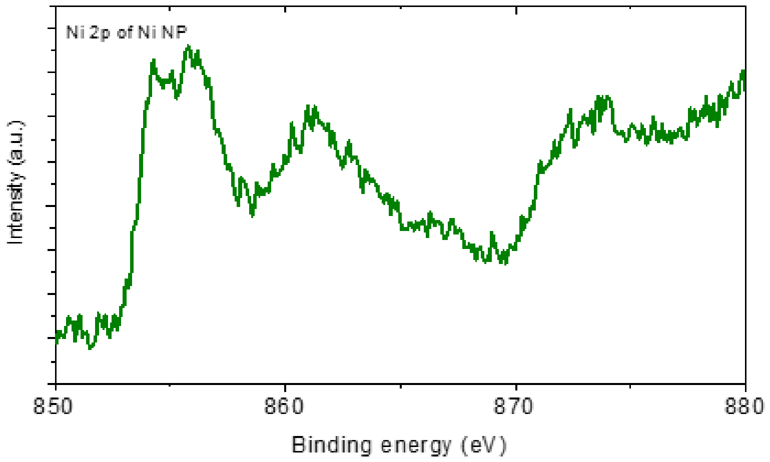

Supplementary Figure 11. **Ni 2p X-ray photoelectron spectroscopy of Ni NP**

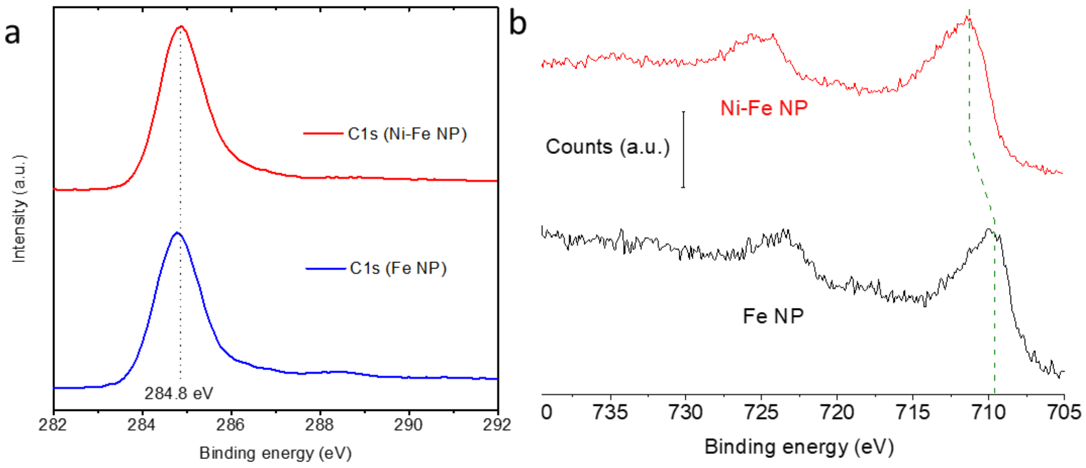

Supplementary Figure 12. C1s XPS scans

(a) Ni-Fe NP and Fe NP; (b) Comparison of high resolution Fe $2p_{3/2}$ XPS scans of Ni-Fe NP and Fe NP.

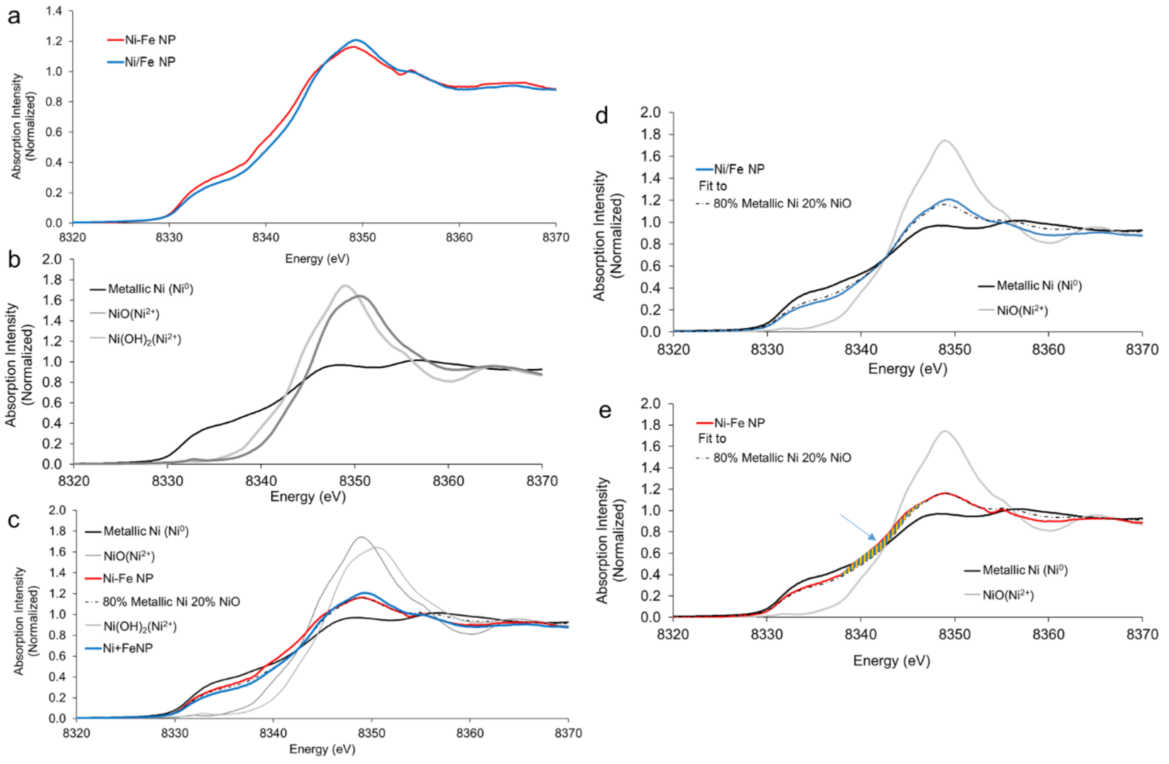

Supplementary Figure 13. Ni K-edge XANES taken on Ni-Fe NP and Ni/Fe NP

a–c show the XANES data and three key references, metallic nickel, nickel oxide and $Ni(OH)_2$ (LDH structure). d–e show the best fit from the reference materials. Arrow indicates place where the linear combination fit is inadequate.

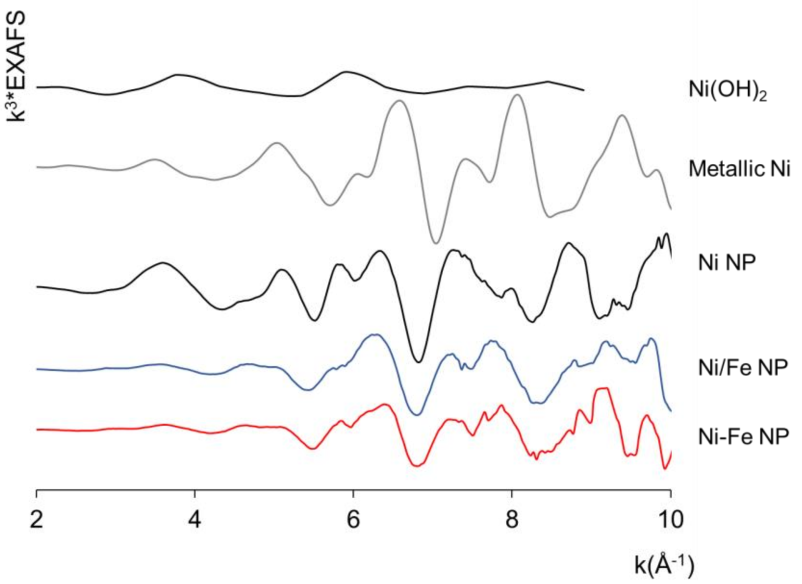

Supplementary Figure 14. EXAFS measurement

Ni K-edge taken on Ni-Fe NP compared to three key references, Ni NP (NiO), Metallic Nickel and $Ni(OH)_2$.

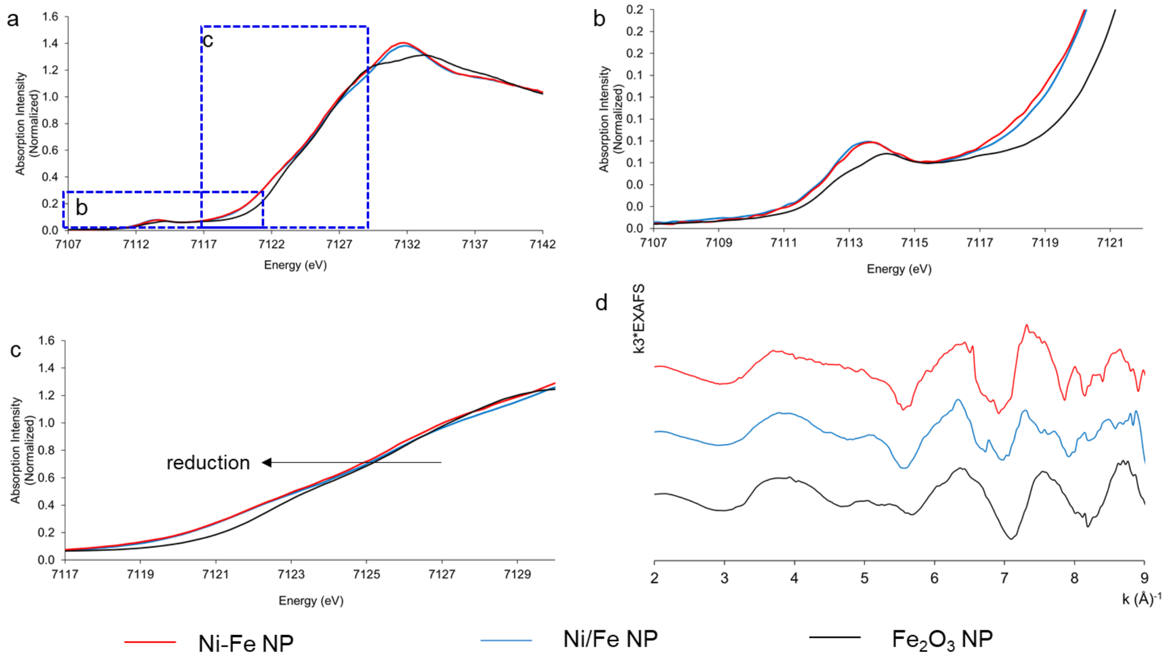

Supplementary Figure 15. Fe K-edge XANES taken on Ni-Fe NP before and after chemical reactions

a–c show the XANES data and the Fe_2O_3 reference material. d shows the EXAFS data taken on the same series of materials.

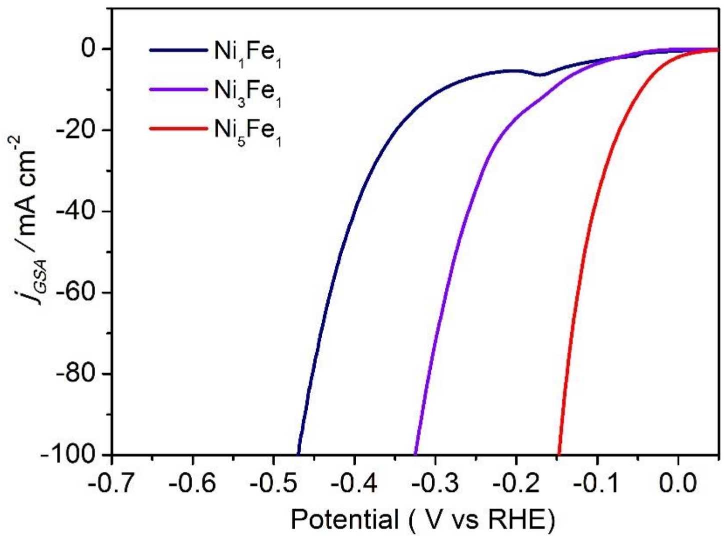

Supplementary Figure 16. Ni-Fe molar ratio optimization

The LSV curves for HER of Ni-Fe NPs with different Ni and Fe molar ratio, the ratio of 5 to 1 (red line) corresponds to Ni-Fe NP.

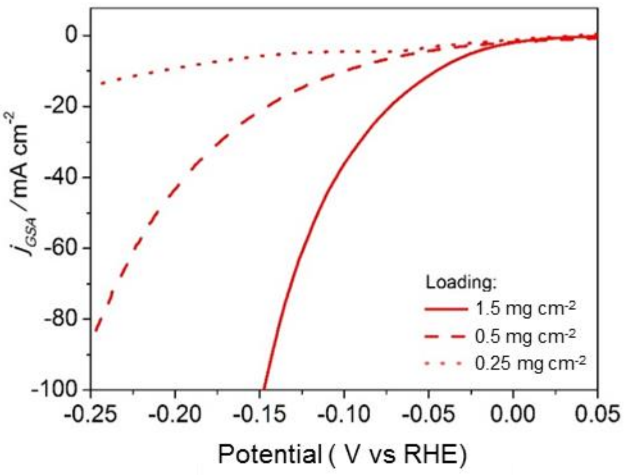

Supplementary Figure 17. The effect of catalyst mass loading on HER LSV curves

LSV curves with HER response from CFP supported Ni-Fe NP with different amount of mass loading (estimated from average atomic mass of Ni and Fe (58.2 g mol^{-1}) from a micellar solution with concentration of 25 mg mL^{-1}).

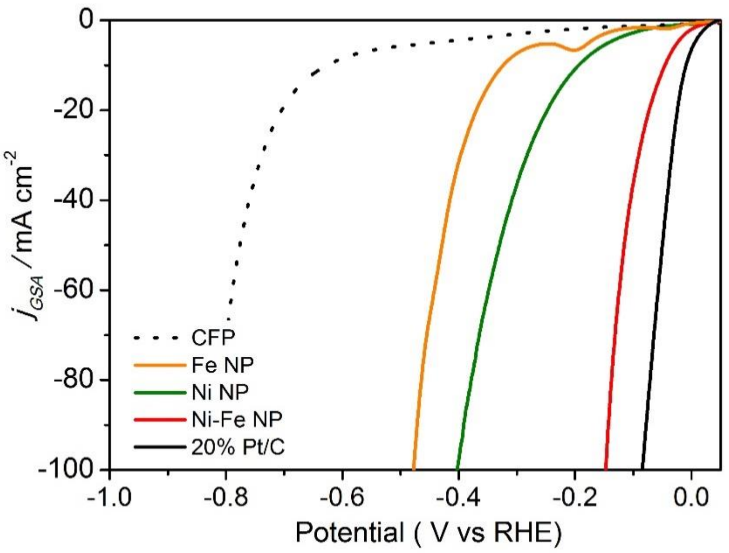

Supplementary Figure 18. **LSV curve for HER with *iR*-correction**

The LSV curves for HER of Ni NP, Fe NP, Ni-Fe NP and 20% Pt/C in 1 M KOH supported on CFP.

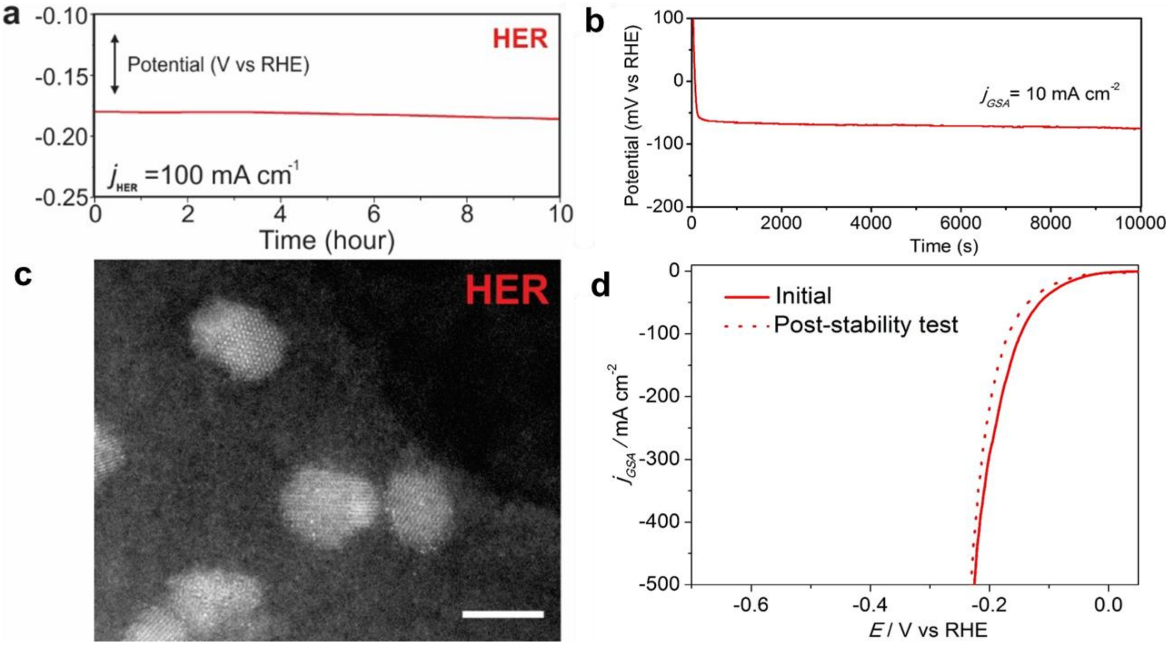

Supplementary Figure 19. Electrochemical HER stability of Ni-Fe NP

(a) The potential (E-t) trace at a constant applied current of 100 mA cm^{-2}, (b) 10 mA cm^{-2}, (c) HAADF-STEM image of the Ni-Fe NP following stablity testing shown in Supplementary Figure 19a-b (scale bar: 5 nm); (d) LSV curves obtained prior (solid line) and after (dashed line) galvanostatic experiments shown in Supplementary Figure 19a–b.

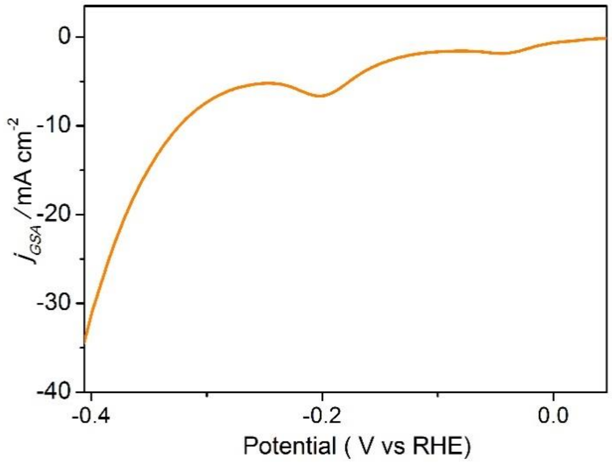

Supplementary Figure 20. Linear sweep voltammetry of Fe NP

LSV curve of Fe NP showing the presence of two-step reduction peaks of Fe.

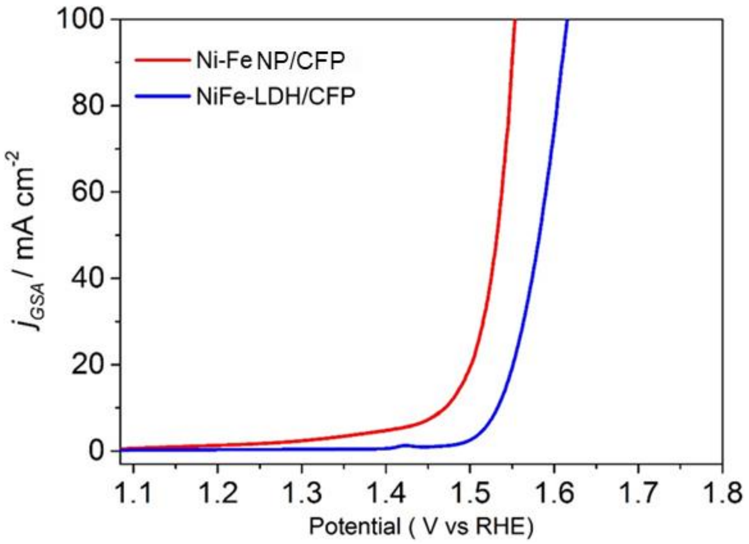

Supplementary Figure 21. LSV of Ni-Fe NPs supported on CFP

LSVs of Ni-Fe NPs supported on CFP for oxygen evolution reaction (OER). As comparison, state-of-art NiFe-LDH was fabricated and its OER performance was compared in 1 M KOH. (Note that Ni oxidation peak was not observed on Ni-Fe NPs anodic polarization scan).

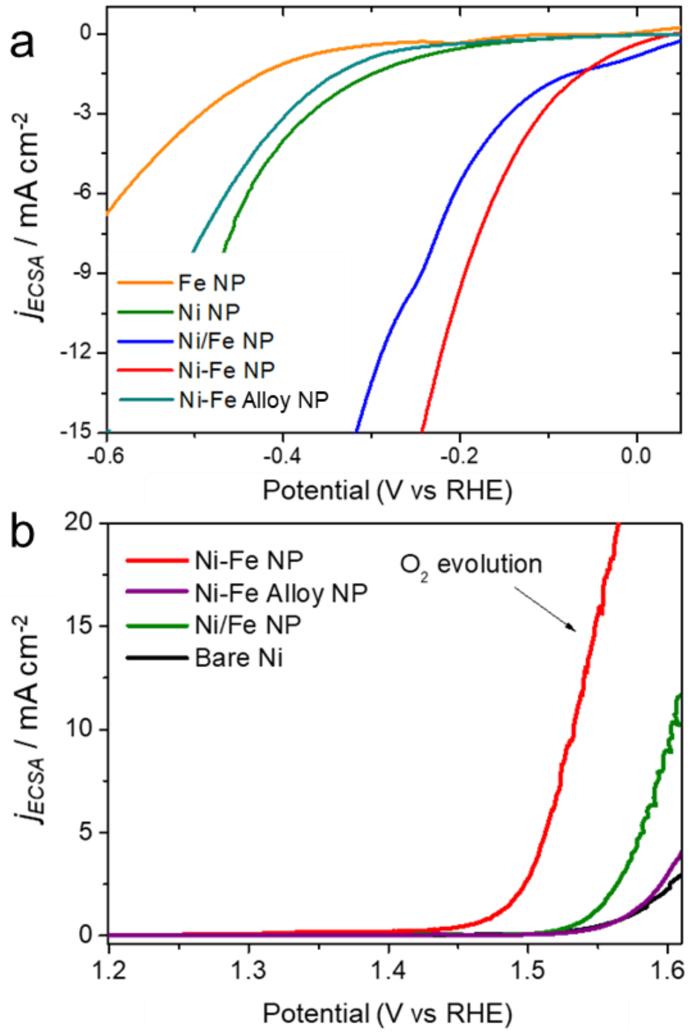

Supplementary Figure 22. **The ECSA normalized HER**

(a) and OER (b) LSV curves. All measured currents were normalized with the measured ECSA shown in Supplementary Table 1.

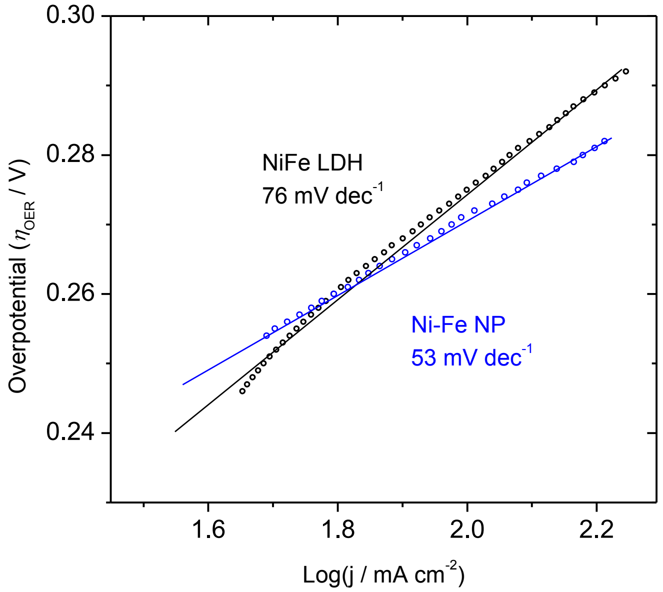

Supplementary Figure 23. OER Tafel plots

Tafel plots derived from the LSV curve in Fig 3c.

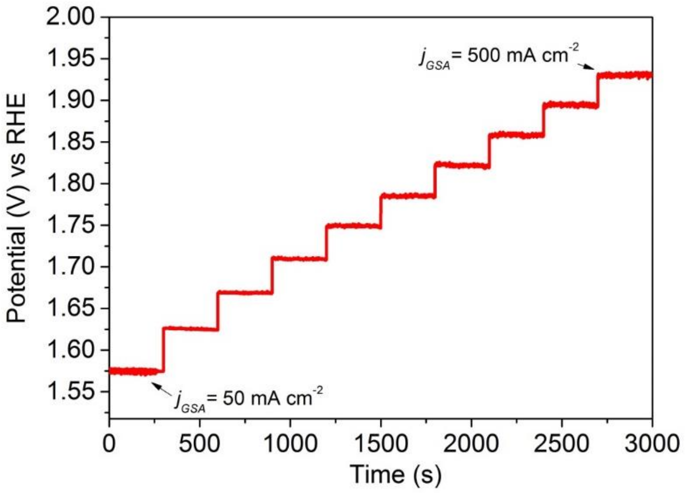

Supplementary Figure 24. OER staircase voltammetry of Ni-Fe NP

Multi-current process obtained with the Ni-Fe NPs on NF in 1 M KOH. The current density started at 50 mA cm^{-2} and finished at 500 mA cm^{-2}, with an increment of 50 mA cm^{-2} every 300 s. (without iR-correction)

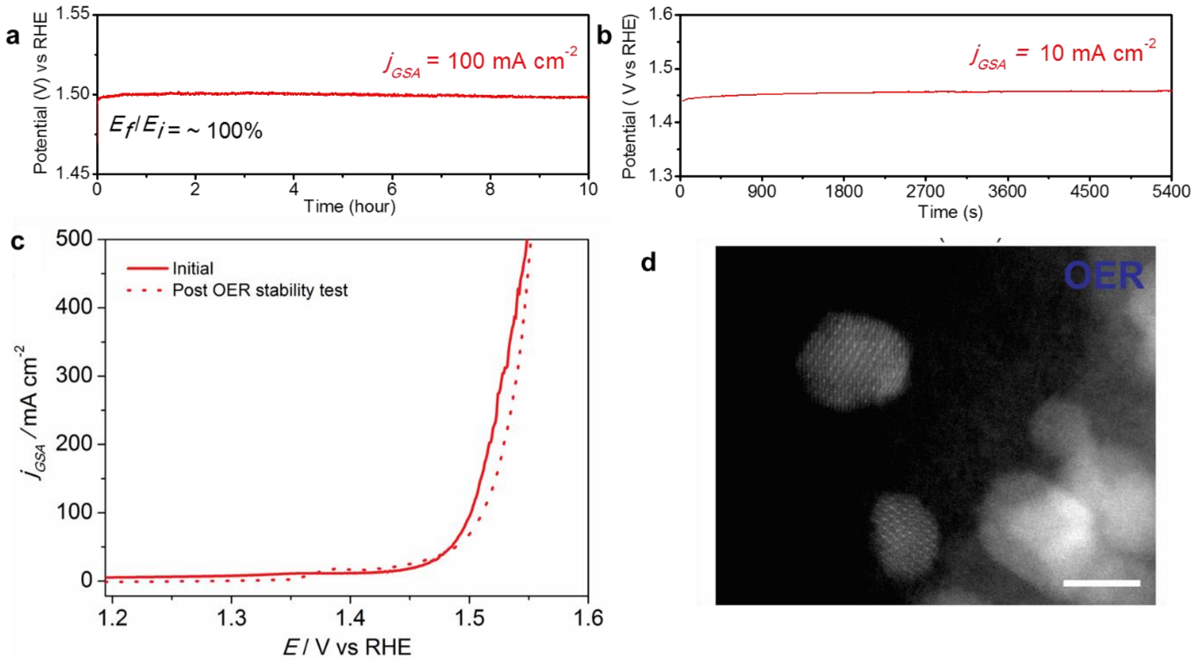

Supplementary Figure 25. Electrochemical OER stability of Ni-Fe NPs

(a) The potential (E-t) trace at a constant applied current of 100 mA cm^{-2}, (b) 10 mA cm^{-2}, (d) LSV curves obtained prior (solid line) and after (dashed line) galvanostatic experiments shown in Supplementary Figure 25a–b; (d) HAADF-STEM image of the Ni-Fe NP following stablity testing shown in Supplementary Figure 25a–b (scale bar: 5 nm).

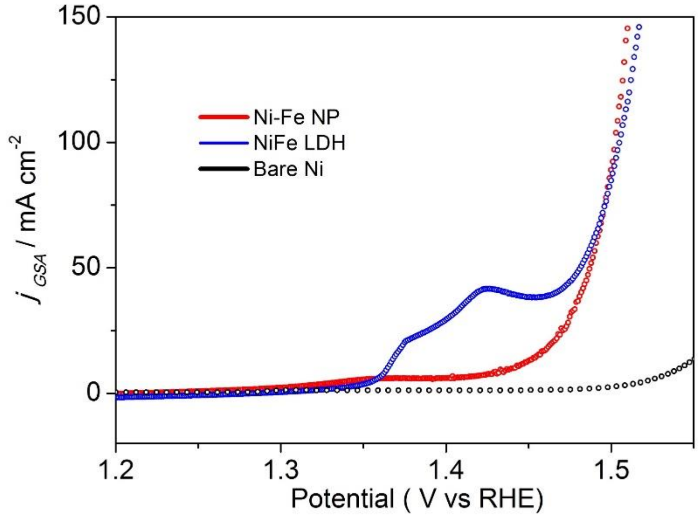

Supplementary Figure 26. LSVs of Ni-Fe NPs on NF for OER

Zoom of LSVs in Fig 3c, to clearly show the Ni oxidation peaks.

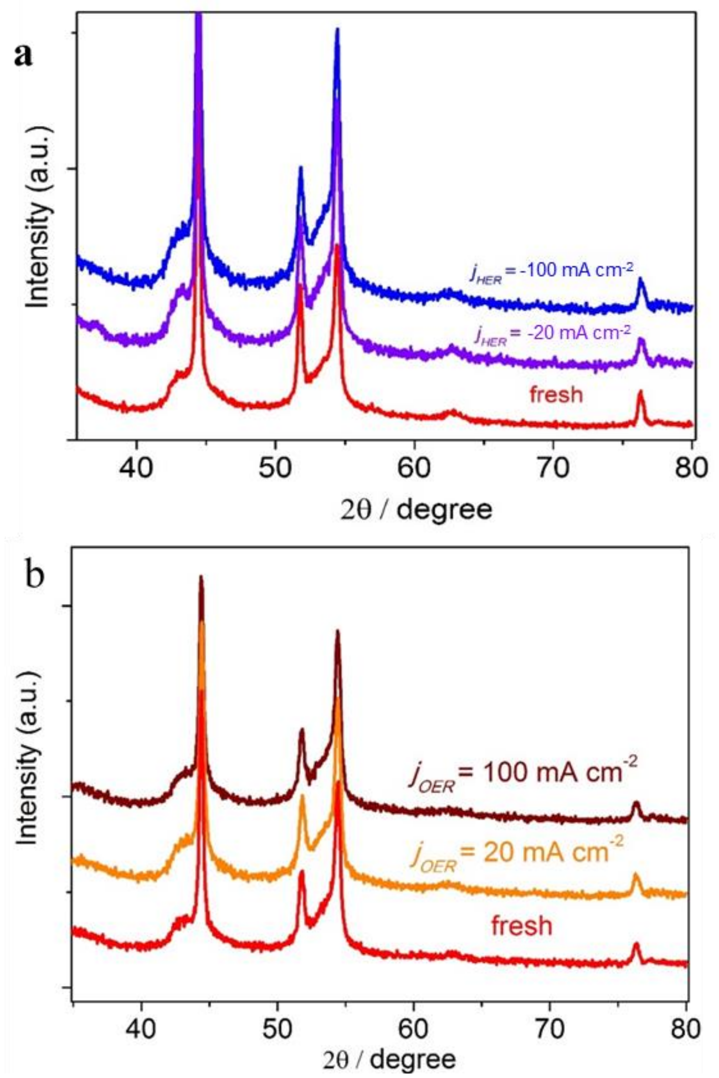

Supplementary Figure 27. XRD characterization of Ni-Fe NPs following HER-OER stability tests.

(a) XRD spectra of Ni-Fe NPs following HER long-term stability (5 hours) at constant *j* of -20 mA cm^{-2} (violet line) and −100 mA cm^{-2} (blue line) (b) XRD spectra of Ni-Fe NPs following long term OER stability (5 hours) at constant *j* of 20 mA cm^{-2} (orange line) and 100 mA cm^{-2} (maroon line).

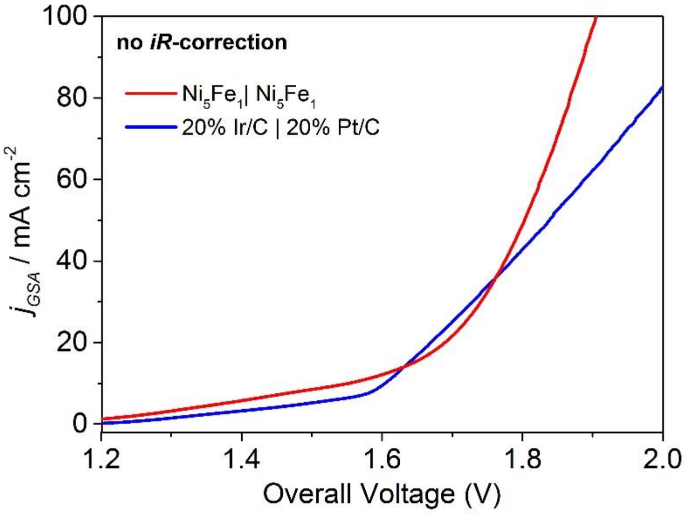

Supplementary Figure 28. **Evaluation of 2-electrodes water electrolyser cell performance (without iR-correction)**

(a) LSV of overall water splitting of Ni-Fe NP (Ni_5Fe_1) cell and Pt/C|Ir/C collected at scan rate of 5 mV s^{-1}.

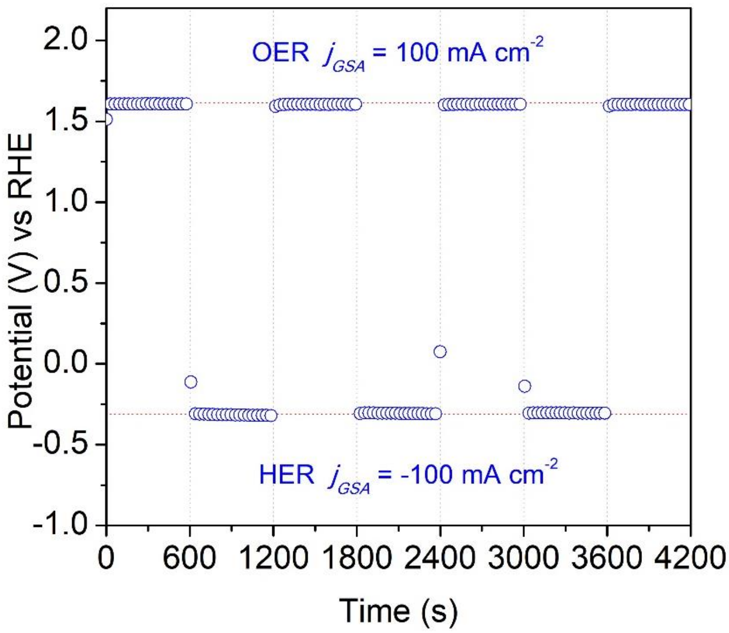

Supplementary Figure 29. **Accelerated degradation test (ADT)**

The ADT of Ni-Fe NP/NF electrode, simulating the power interruption induced electrode depolarization.

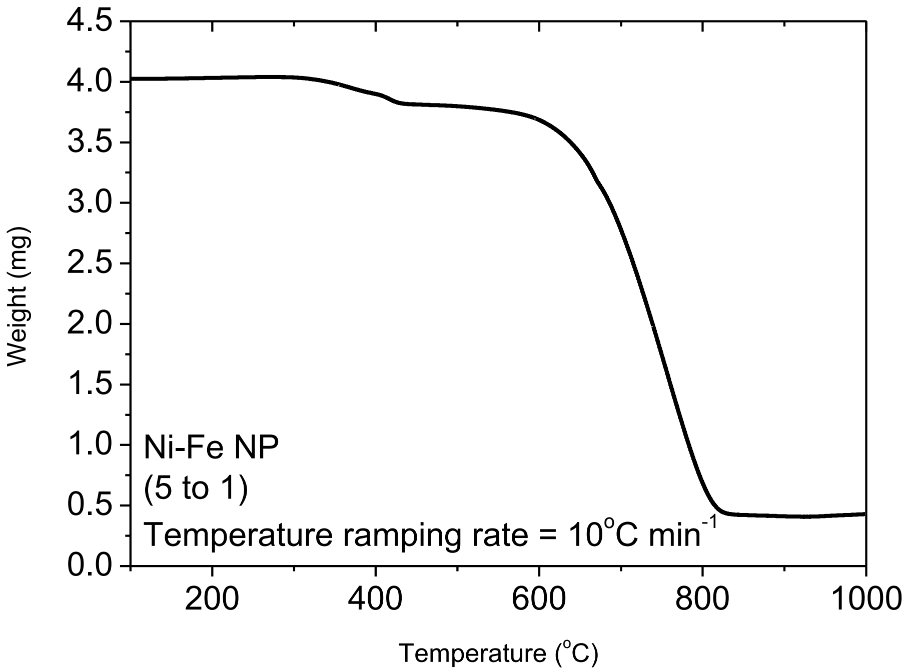

Supplementary Figure 30. Thermogravimetric analysis (TGA)

TGA of Ni-Fe NP/CFP (GSA: 0.25 cm^2). Experimental conditions: measurement range: 100°C–1000°C, Temperature ramping rate: 10°C min^{-1}, ambient atmosphere.

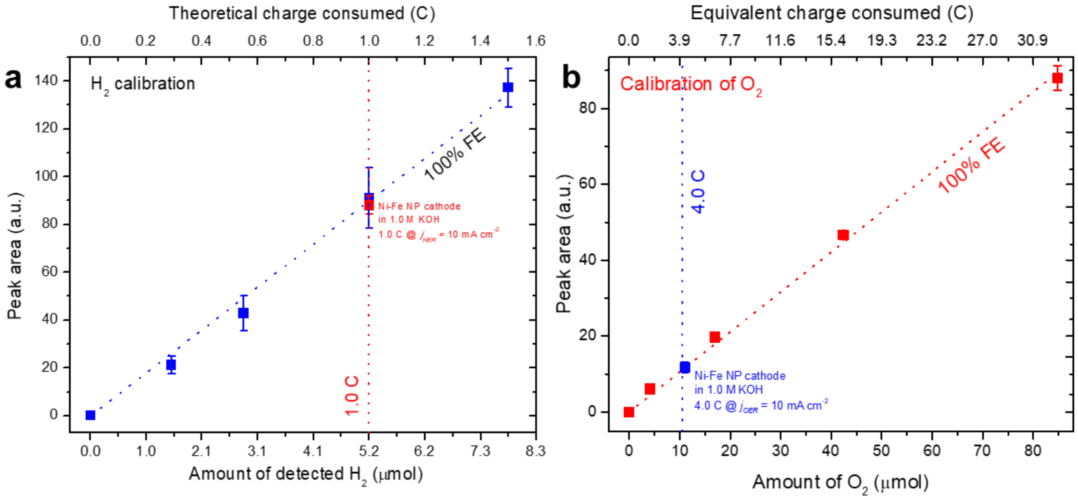

Supplementary Figure 31. Faradaic efficiency (FE) measurement with gas chromatography

(a) H_2 gas chromatographic calibration plot was made from a series of different H_2 amounts produced by HER on a Pt electrode in 50 mM H_2SO_4, which is known to exhibit FE of 100%. The superposed red plot corresponds to a H_2 amount generated using 1.0 C on Ni-Fe NP at j_{HER} = 10 mA cm^{-2}, showing a Ni-Fe NP HER FE of 96.8 ± 4.2%, (n = 3). (b) O_2 calibration plot was made by a series of dilution of air (21% O_2) with high purity N_2, the superposed blue plot corresponds to a O_2 amount generated using 4.0 C on Ni-Fe NP at j_{OER} = 10 mA cm^{-2}, showing a Ni-Fe NP OER FE of 106 ± 14%, (n = 3). Each of the calibration points are measured with at least n = 2. The best fit lines in both of calibration curve correspond to theoretical 100% FE.

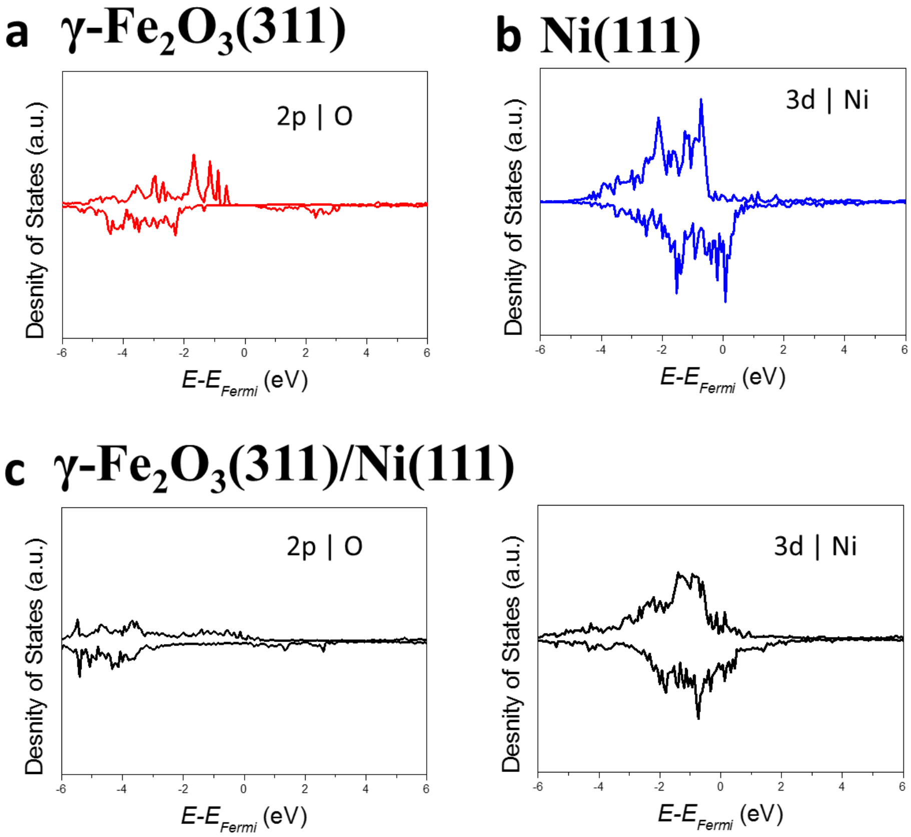

Supplementary Figure 32. Partial Density of states of surface O and Ni atoms

(a) 2p states of O atom in the γ-Fe_2O_3(311) surface, (b) 3d states of Ni atom in the Ni(111) surface, (c) 2p states of O atom and 3d states of Ni atom at the interface of the Ni-Fe heterojunction.

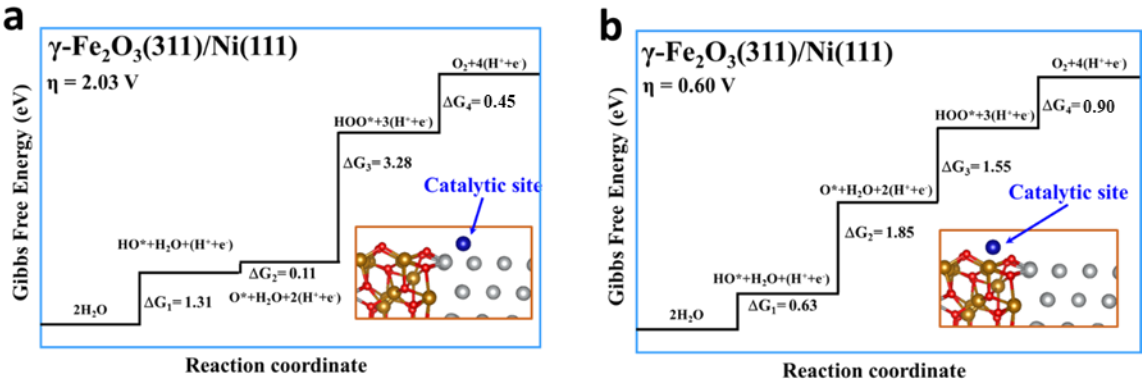

Supplementary Figure 33. Standard free energy diagram of the OER process

(a) on the Ni side of the Ni-Fe heterojunction, (b) on the Fe_2O_3 side of the Ni-Fe heterojunction.

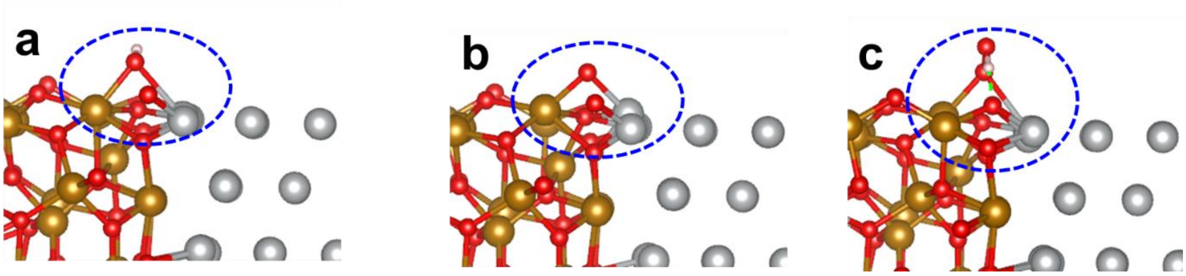

Supplementary Figure 34. **The optimized structure of the adsorption of intermediates** (a) OH, (b) O and (c) OOH. The green dash line shown in (c) indicates the formation of the hydrogen bond between the H atom in the OOH and the O atom at the Ni-Fe_2O_3 interface, which can stabilize the transition state of OOH.

Supplementary Tables

Supplementary Table 1. Electrochemically active surface area (ECSA) determination of the NF supported nanoparticles.

Samples	Weight of substrate (g)	GSA[a]	C_{DL} (mF)			ECSA	RF[b]
			Anodic	Cathodic	Average		
Ni-Fe NP	0.070	0.95	1.3	1.2	1.25	31.0	32.5
Ni-Fe alloy NP	0.054	0.74	3.1	3.1	3.1	77.5	105.2
Ni/Fe NP	0.059	0.81	0.4	0.4	0.4	10.0	12.4
Nickel Foam	0.064	0.87	0.9	0.9	0.9	22.4	25.6
**CFP Substrate**							
Ni-Fe NP	0.02	1.5	0.22	0.22	0.22	5.5	3.7
Ni/Fe NP	0.02	1.5	0.12	0.12	0.12	3	2
Ni NP	0.02	1.5	0.58	0.56	0.57	14.2	9.5
Fe NP	0.02	1.5	0.48	0.48	0.48	12	8
Ni-Fe alloy NP	0.02	1.5	0.62	0.64	0.63	15.8	10.5
CFP	0.02	1.5	0.18	0.18	0.18	4.5	3

[a]geometric surface area; [b]roughness factor

Supplementary Table 2. Comparison of HER catalytic activity with recently reported HER active from non-precious materials.

Catalyst	η(mV) 10 mA cm^{-2}	η(mV) 20 mA cm^{-2}	η(mV) 100 mA cm^{-2}	Tafel / mV dec^{-1}	Substrate	Loading / mg cm^{-2}	Electrolyte	Year	Ref.
**Ni-Fe NPs (Ni_5Fe_1)**	−46	−73	−147	58	CFP	1.5	1.0 M KOH	This work	
**Ni_3Fe_1**	−154	−214	−323	124	CFP				
**Ni_1Fe_1**	−288	−348	−470	251	CFP				
**Ni NP**	−190	−248	−402	167	CFP				
**Fe NP**	−320	−370	−476	186	CFP				
**Metal Chalcogenides/Nitrides/Phosphides**									
**Ni_3FeN/rGO***	−94	−125	−190	90	NF	0.5	1.0 M KOH	2018	1
**$Ni_{11}(HPO_3)_8(OH)_6$***	−121	n.a.	−274	102	NF	3	1.0 M KOH	2018	2
**$Ni(OH)_2$-Ni_3N***	n.a.	−72	−181	86	Ti Mesh	3.2	1.0 M KOH	2018	3
**N/Ni_3S_2**	−155	−190	−320	152	NF	0.6	1.0 M KOH	2018	4
**Ni_3S_2**	−240	-290	n.a.	113	NF	0.6	1.0 M KOH	2018	4
**$Ni_{0.89}Co_{0.11}Se_2$ MNSN**	−85	−100	−150	52	NF	2.23	1.0 M KOH	2017	5
**Janus Co/CoP***	−193	−280	n.a.	64	NF	0.22	1.0 M KOH	2017	6
**h-NiS_x**	−60	n.a.	n.a.	99	NF	[c]Enhanced area	1.0 M KOH	2016	7
**Ni_5P_4**	−49	n.a.	−202	98	n.a.	pellet	1.0 M KOH	2016	8
**CoSe/NiFe LDH**	−260	−348	n.a.	125	NF	4	1.0 M KOH	2016	9
**Metal oxides / Layered double hydroxide**									
**$Ni_3Fe(OH)_9$/Ni_3Fe***	−217	n.a.	−355	83	NF	0.7	0.1 M KOH	2018	10
**NiFe-MOF***	−134	n.a.	n.a.	n.a.	NF	0.3	0.1 M KOH	2017	11
**Cu@Ni-Fe LDH***	−116	−130	−192		Cu foam	2.2	1.0 M KOH	2017	12
**NiFe/$NiCo_2O_4$**	−105	−140	−202	88	NF	n.a.	1.0 M KOH	2016	13
**np-CuTi**	−50	−75	n.a.	110	porous Cu	n.a.	0.1 M KOH	2015	14
**NiO/Ni-CNT**	n.a.	n.a.	−100	51	NF	8	1.0 M KOH	2014	15
**NiFe-LDH**	−210	−250	n.a.	n.a.	NF	n.a	0.1 M KOH	2014	16
**Ni-Mo nanopowder**	n.a.	−70[a]	−130[b]	n.a.	Ti	13.4	2.0 M KOH	2013	17

***** CFP = carbon fibre paper, GC = glassy carbon electrode, n.a. = data was not produced in the literature. All of the data compared in this table is based on catalytic activity (LSV) of materials taken in 1 M KOH, unless otherwise specified,

Supplementary Table 3. ICP-MS analysis on dissolved Ni and Fe during HER stability testing.

Samples	Fe (μg L^{-1})	Ni (μg L^{-1})
HER @ j = −50 mA cm^{-2}, 1 h	$< 5^{*}$	$< 5^{*}$
HER @ j = −50 mA cm^{-2}, 10 h	70^{*}	$< 5^{*}$

**all of the reported amount has been background substracted.*

Supplementary Table 4. Comparison of OER catalytic activity with recently reported OER active from non-precious materials.

**Catalyst**	***n*(mV)**			**Tafel / mV dec**	**Substrate**	**Loading / mg cm^{-2}**	**Electrolyte**	**Year**	**Ref.**
	**10 mA cm^{-2}**	**20 mA cm^{-2}**	**100 mA cm^{-2}**						
**Ni-Fe NPs**	210	230	270	53	NF	2.5	1.0 M KOH	This work	
**Metal oxides / Layered double hydroxides**									
**Ni3FE(OH)9/Ni3Fe**	228	250	295	28	NF	0.7	0.1 M KOH	2018	10
**Cu@Ni-Fe LDH**	199	n.a.	281	28	Cu foam	2.2	1.0 M KOH	2017	12
**NiFe-MOF**	240	n.a.	300	34	NF	0.3	0.1 M KOH	2017	11
**NiFe (electrodeposition)**	215	n.a.	270*	33	NF	electrodepositon	1.0 M KOH	2015	18
**NiFeO$_x$-Li induced**	230	~250	>270	31.5	CFP	Li-induced activation	1.0 M KOH	2015	19
**Ni-Fe LDH (hydrothermal)**	230*	280**	n.a.	50	NF	hydrothermal	1.0 M KOH	2014	20
**Metal Chalcogenides/Nitrides/Phosphides**									
**Ni_3FeN/rGO**	270	283	320	54	NF	0.5	1.0 M KOH	2018	1
**$Ni_{11}(HPO_3)_8(OH)_6$**	232	270	362	91	NF	3	1.0 M KOH	2018	2
**Janus Co/CoP**	340	415	590	79.5	NF	0.22	1.0 M KOH	2017	6
**h-NiS_x**	180	183	217	96	NF	Enhanced area	1.0 M KOH	2016	7
**CoSe/NiFe LDH**	pre-oxidation peak	pre-oxidation peak	260	57	NF	4	1.0 M KOH	2016	9
**Ni3S2**	260	275	n.a.	n.a.	NF	1.6	1.0 M KOH	2015	21

Supplementary Table 5. ICP-MS analysis on dissolved Ni and Fe during OER stability testing.

Samples	Fe (μg L^{-1})	Ni (μg L^{-1})
OER @ j = 50 mA cm^{-2}, 1 h	< 5*	< 5*
OER @ j = 50 mA cm^{-2}, 10 h	< 5*	14.1*

**all of the reported amount has been background substracted.*

Supplementary Table 6. Comparison of recently reported alkaline water electrolyzer catalyst in 2-electrodes configuration.

Anode	Cathode	Substrate	E_{cell} @ 10 mA cm^{-2}	$J_{1.8V}$ / mA cm^{-2}	*iR*-correction	Reference
**Ni-Fe**	Ni-Fe	Ni_{foam}	1.47 V	150	Yes	This work
**Ni-Fe**	Ni-Fe	Ni_{foam}	1.55 V	50	No	This work
**Ir/C**	Pt/C	Ni_{foam}	1.55 V	64	Yes	This work
**Ir/C**	Pt/C	Ni_{foam}	1.6 V	43	No	This work
**NiFe-LDH**	NiFe-LDH	Ni_{foam}	1.70 V	n.a.	No	16
**Co-P/NC**	Co-P/NC	GC	1.70 V	~40	Yes	22
**Co-P**	Co-P	Cu_{foil}	1.64 V	100**	n.a.	23
**NiFeO**	NiFeO	CNF	1.51 V	80	Yes	19
**NiFe-LDH**	NiO/Ni-CNT	Ni_{foam}	~1.50 V for 20 mA cm^{-2}	n.a.	Yes	15
**CoMnO/CN**	CoMnO/CN	Ni_{foam}	1.50 V	108	Yes	24
**Co_2B**	Co_2B	CC	1.81 V	<10	n.a.	25
**h-NiSx**	h-NiSx	Ni_{foam}	1.47 V	n.a.	Yes	7
**e-ICLDH @GDY/NF**	e-ICLDH @GDY/NF	Ni_{foam}	1.43 V	~500	No	26
**FeP/Ni_2P**	**FeP/Ni_2P**	Ni_{foam}	1.42 V	>1000	n.a.	27

***** GC = glassy carbon electrode, n.a. = data was not produced in the literature. All of the data compared in this table is based on catalytic activity (LSV) of materials taken in 1 M KOH, unless otherwise specified.

Supplementary Table 7. Bader charge of O and Ni atoms in the γ-Fe_2O_3(311), Ni(111) and their heterojunction.

	**γ-Fe_2O_3(311)**	**Ni(111)**	**heterojunction**
**O**	−1.0		−1.1
**Ni**		0.0	0.4

Supplementary Table 8. Binding energy of O ($E_b = E_{tot} – E_{surf} – E_o$, where the E_{tot}, E_{surf} and E_o represent the energies of the surface with the adsorbed O atom, bare surface and isolated O atom, respectively. The more negative values means a stronger binding strength) on the γ-Fe_2O_3(311), Ni(111) and their heterojunction.

	**γ-Fe_2O_3(311)**	**Ni(111)**	**Ni-Fe heterojunction**
***E_b* (eV)**	−3.33	−5.34	−3.79

Supplementary Methods

X-ray diffraction spectroscopy (XRD)

XRD measurements were performed with PANalytical X'Pert Empyrean instrument equipped with standard Cu anode, K-α wavelength = 1.54 Å. The typical scan range (2θ) was 10° to 80°, collected with step size of 0.039^{o} s^{-1}.

X-ray photoelectron spectroscopy (XPS)

XPS measurements were performed with Thermo ESCALAB250i X-ray photoelectron spectrometer. The high-resolution measurements were conducted with 500 μm spot size and a pass energy of 20 eV. To ensure the results consistency, the scan was performed at 4 different spots. The binding energies reported in this study were calibrated to adventitious hydrocarbon at 284.8 eV.

X-ray absorption spectroscopy (XAS)

Ni and Fe K-edge XAS spectra were recorded on the multipole wiggler XAS beam-line 12 ID at the Australian Synchrotron, in operational mode 1. The beam energy was 3.0 GeV and the maximum beam current was 200 mA. Reference samples for the transmission X-ray experiment were prepared by grinding the sample together with boron nitride and loading into a 1 mm thin Al spacer and sealed with 63.5 μM Kapton tape. Data was taken in He/N_2-filled ionisation chambers. Data on electrodes was taken directly on the carbon fibre matrix in fluorescence mode using a 100 element fluorescence detector. Data was energy calibrated against the first inflection point of the Fe foil to 7112 eV; and Nickel 8330 eV. EXAFS E0 values used were 7130 eV for Fe and 8350 eV for Ni. XAS data was analysed using a combination of PySpline[2] and Microsoft Excel[3] for background subtractions, and Artemis[4-5] for EXAFS fitting.

Transmission electron microscopy (TEM)

TEM was carried out with Phillips CM 200 microscope. To prepare TEM samples, the CFP supported catalyst was transferred to Cu-grid by physically scratching the electrode using a sharp knife. The resulting powder was dispersed in absolute ethanol by ultrasonication for 15 mins. The resulting mixture was then drop-casted onto Cu-grid and dried in room temperature.

Scanning transmission electron microscopy (STEM)

STEM was carried out with JEOL JEM-ARM200F equipped with aberration corrected 200 kV electron beam source. To prepare STEM samples, the CFP supported Ni-Fe NPs was transferred to Cu-grid coated with holey carbon by physically scratching the electron using a sharp knife. The resulting powder was dispersed in absolute ethanol by ultrasonication for 15 mins. The resulting mixture was then drop-casted onto Cu-grid and dried in room temperature.

Computational Methodology

All spin-polarized density-functional theory (DFT) computations were carried out using the Vienna *ab initio* simulation package (VASP) based on the projector augmented wave (PAW) method[28,29]. Electron-ion interactions were described using standard PAW potentials, with valence configurations of $3s^2 3p^6 4s^2 3d^6$ for Fe (Fe_sv_GW), $3s^2 3p^6 4s^2 3d^8$ for Ni (Ni_sv_GW), $2s^2 2p^4$ for O (O_GW_new), and $1s^1$ for H (H_GW). A plane-wave basis set was employed to expand the smooth part of wave functions with a cut-off kinetic energy of 520 eV. For the electron-electron exchange and correlation interactions, the functional parameterized by Perdew-Burke-Ernzerhhof (PBE),[31] a form of the general gradient approximation (GGA), was used throughout. Due to insufficient consideration of the on-site Columbic repulsion between the Fe d electrons in the oxide, DFT might fail to describe the

electronic structure of γ-Fe_2O_3. To overcome this shortcoming, the GGA+U approach was used with U−J = 3.0 eV for the Fe atoms[31].

To study the mechanistic chemistry of surface reactions, the γ-Fe_2O_3 (311) surface was modeled using a slab with eight oxygen layers. And the Ni (111) surface was modelled using a slab with six nickel layers. To build the interface model of the Ni-Fe heterojunction, a γ-Fe_2O_3 (311) (1 × 1) unit cell is combined with a Ni(111) (4 × 2√2) unit cell since their lattices constants along the *x* direction are close to each other (10.20 Å and 9.96 Å for the γ-Fe_2O_3 (311) (1 × 1) and Ni(111) (4 × 2√2), respectively), which may be one of the driving forces to the formation of the heterojunction due to the small strain at the interface. As such, the lattice constants of the interface model along the *x* axial is initially set as 10.0 Å. And the lattice constants *a* and *b* were further optimized to get the stable interface configuration. A sufficiently large vacuum region of 15 Å was used for all the systems to ensure the periodic images to be well separated. During the geometry optimizations, all the atoms were allowed to relax.

The Brillouin-zone integrations were conducted using Monkhorst-Pack grids of special points. A gamma-centered (2 × 4 × 1), (5 × 5 × 1) and (2 × 2 × 1) *k*-point grids were used for the γ-Fe_2O_3 (311) (1 × 1), Ni (111) (2 × 2) surface cells and the interface model. The H_2, O_2 and H_2O molecules were calculated in a 20 × 20 × 20 $Å^3$ box. The convergence criterion for the electronic self-consistent loop was set to 10^{-4} eV. And the atomic structures were optimized until the residual forces were below 0.01 eV $Å^{-1}$. Based on DFT results, the magnetic moments for Fe^{3+} and Ni^0 metals are around 4.0 and 0.7 μ_B, respectively, which indicates that the Fe^{3+} has a high spin state with five unpaired electrons. The antiferromagnetic (AFM) structures were used for the γ-Fe_2O_3 and the interface models based on the superexchange model[32].

Theoretical overpotential of hydrogen evolution reaction (HER)

Under the standard condition, the overall HER pathway includes two steps: first, adsorption of hydrogen on the catalytic site (*) from the initial state ($H^+ + e^- + *$), second, release the product hydrogen ($1/2H_2$). The total energies of $H^+ + e^-$ and $1/2H_2$ are equal. Therefore, the Gibbs free energy of the adsorption of the intermediate hydrogen on a catalyst (ΔG_H) is the key descriptor of the HER activity of the catalyst and is obtained by Supplementary Equation 1:

$$\Delta G_H = \Delta E_H + \Delta ZPE - T\Delta S \qquad (1)$$

where ΔE_H, ΔZPE and ΔS are the binding energy, zero point energy change and entropy change of H adsorption, respectively. Herein, the value of $\Delta ZPE - T\Delta S$ is about 0.24 eV calculated by Norskov et al.,[33] And ΔE_H is calculated using the Supplementary Equation 2:

$$\Delta E_H = E_{surf+H} - E_{surf} - ½E_{H2} \qquad (2)$$

The E_{surf+H} is the total energy of the system with one adsorbed H atom in each unit cell. And E_{surf} and E_{H2} represent the energy of the bare surface and H_2 gas molecule, respectively.

Theoretical overpotential of oxygen evolution reaction (OER) method

In an acidic solution, there are four OER fundamental steps for OERs:

$$2H_2O \leftrightarrow \mathbf{OH*} + H^+ + e^- + H_2O \leftrightarrow \mathbf{O*} + 2H^+ + 2e^- + H_2O \leftrightarrow \mathbf{OOH*} + 3H^+ + 3e^- \leftrightarrow O_2 + 4H^+ + 4e^-$$

In an alkaline electrolyte, the anode reactions could be written as:

$$4OH^- \leftrightarrow \mathbf{OH*} + 3OH^- + e^- \leftrightarrow \mathbf{O*} + 2OH^- + 2e^- + H_2O \leftrightarrow \mathbf{OOH*} + OH^- + 3e^- \leftrightarrow O_2 + 2H_2O + 4e^-$$

As such, the Gibbs free energy of the adsorption of intermediates including OH*, O* and OOH* can be used to calculate the change of Gibbs free energy of each fundamental step (ΔG_n, n =1–4), which is approximately independent to the pH value of the solution.

The similar method ($\Delta G = \Delta E + \Delta ZPE - T\Delta S$) also developed by Norskov et al.,[34] to calculate the Gibbs free energy of the adsorption of intermediates including OH*, O* and OOH*. The

entropies and zero point energies (ZPEs) used in the construction of the reaction free energies are obtained from the previous results.[34] ΔG_n is finally calculated using the equations:

$\Delta G_1 = \Delta G_{OH^*}$, $\Delta G_2 = \Delta G_{O^*} - \Delta G_{OH^*}$, $\Delta G_3 = \Delta G_{OOH^*} - \Delta G_{O^*}$, $\Delta G_4 = -E_{O2} + 2E_{H2} - 2E_{H2O} - \Delta G_{OOH^*}$

E_{H2}, E_{O2} *and* E_{H2O} represent the energy of the H_2, O_2 and H_2O gas molecules, respectively.

Supplementary Notes

Determination of electrode surface area by mass

To ensure reproducibility of geometric surface area (GSA) of NF, the reported GSA were measured by mass, the mass to area conversion is carried out by the following equation:

Known:

Bulk density = 0.45 g cm^{-3}

NF thickness = 0.16 cm

NF weight used to prepare electrodes = 0.22 g

GSA of NF = Volume of NF / thickness of NF (Volume = SA × thickness)

Therefore,

GSA of NF = (Mass of NF / Bulk density) / NF thickness

GSA of NF = (0.22 g / 0.45 g cm^{-3}) / 0.16 cm = 3 cm^2

Determination of catalyst mass loading

Since a fixed amount of 6 mmol of metals were used in the preparation of metal-oleate complexes (in 14 mL of hexane), the metal mass loading of the prepared electrode can be determined by the following calculation:

Metal ions concentration in hexane: 0.43 mmols mL^{-1}

Desired metal loading (m_l) on CFP: 1.5 mg cm^{-2}

Desired metal loading (m_l) on NF: 2.5 mg cm^{-2}

Hence,

Drop-cast volume/cm^2 = (m_l / Atomic weight of metal) / 0.43 mmols mL^{-1}

Determination of electrochemically active surface area

To determine the ECSA of the characterized materials, double layer capacitance values of the different materials have to be first determined.[35] Supplementary Figure 2 shows the cyclic voltammetries (CV) collected using the nanoparticles in comparison supported on NF in 1.0

M KOH. The switching potentials of the CVs were set to be −0.12 and −0.02 V, corresponding to the cathodic and anodic limits, respectively and a center potential of −0.07 V corresponding to the open circuit potential (OCP). The CVs were collected at different scan rates (v) of 0.01, 0.025, 0.05, 0.1 and 0.2 V s^{-1}, which allow the determination of double-layer capacitance (C_{DL}) from Supplementary Equations 3–4:

$$I = vC_{DL} \qquad (3)$$

$$ECSA = C_{DL}/Cs \qquad (4)$$

With *Cs* being the the specific capacitance of typical metal nanoparticle catalyst of 0.04 mF cm^{-2}. These measured values are summarized in Supplementary Table 1.

Determination of turn over frequency (TOF)

The TOF of Ni-Fe NP for OER using the following formula:

TOF = $j \times A / (n \times F \times m)$

OER TOF was calculated based on the recorded current density at an η_{OER} = 350 mV and HER TOF was calculated based on the recorded current density at an η_{HER} = 200 mV, *n* is the number of electron transferred in the reaction per unit of products (2 for H_2 and 4 for O_2), *F* is the Faraday's constant (96,485 C mol^{-1}) and *m* is the number of moles of the Ni and Fe in the electrode.

Supplementary References

1. Gu, Y. *et al.* Electronic structure tuning in Ni_3FeN/r-GO Aerogel toward bifunctional electrocatalyst for overall water splitting. *ACS Nano* **12**, 245–253 (2018).
2. Menezes, P. W. *et al.* A structurally versatile nickel phosphite acting as a robust bifunctional electrocatalyst for overall water splitting. *Energy Environ. Sci.* **11**, 1287–1298 (2018).
3. Gao, M., Chen, L., Zhang, Z., Sun, X. & Zhang, S. Interface engineering of the $Ni(OH)_2$–Ni_3N nanoarray heterostructure for the alkaline hydrogen evolution reaction. *J. Mater. Chem. A* **6**, 833–836 (2018).
4. Kou, T. *et al.* Theoretical and experimental insight into the effect of nitrogen doping on hydrogen evolution activity of Ni_3S_2 in alkaline medium. *Adv. Energy Mater.* **8**, 1703538 (2018).
5. Liu, B. *et al.* Nickel–Cobalt diselenide 3D mesoporous nanosheet networks supported on Ni foam: an all-pH highly efficient integrated electrocatalyst for hydrogen evolution. *Adv. Mater.* **29**, 1606521 (2017).
6. Xue, Z.-H. *et al.* Janus Co/CoP nanoparticles as efficient Mott–Schottky electrocatalysts for overall water splitting in wide pH range. *Adv. Energy Mater.* **7**, 1602355 (2017).
7. You, B. & Sun, Y. Hierarchically porous nickel sulfide multifunctional superstructures. *Adv. Energy Mater.* **6**, 1502333 (2016).
8. Laursen, A. B. *et al.* Nanocrystalline Ni_5P_4: a hydrogen evolution electrocatalyst of exceptional efficiency in both alkaline and acidic media. *Energy Environ. Sci.* **8**, 1027–1034 (2015).
9. Hou, Y. *et al.* Vertically oriented cobalt selenide/NiFe layered-double-hydroxide nanosheets supported on exfoliated graphene foil: an efficient 3D electrode for overall water splitting. *Energy Environ. Sci.* **9**, 478–483 (2016).
10. Xiao, C., Zhang, X., Li, S.-n., Suryanto, B. H. R. & MacFarlane, D. R. In-situ synthesis of core-shell $Ni_3Fe(OH)_9$/Ni_3Fe hybrid nanostructures as highly active and stable bifunctional catalysts for water electrolysis. *ACS Appl. Energy Mater.***1**, 986–992 (2018).
11. Duan, J., Chen, S. & Zhao, C. Ultrathin metal-organic framework array for efficient electrocatalytic water splitting. *Nat. Commun.* **8**, 15341 (2017).
12. Yu, L. *et al.* Cu nanowires shelled with NiFe layered double hydroxide nanosheets as bifunctional electrocatalysts for overall water splitting. *Energy Environ. Sci.* **10**, 1820–1827 (2017).
13. Xiao, C., Li, Y., Lu, X. & Zhao, C. Bifunctional porous NiFe/$NiCo_2O_4$/Ni foam electrodes with triple hierarchy and double synergies for efficient whole cell water splitting. *Adv. Funct. Mater.* **26**, 3515–3523 (2016).
14. Lu, Q. *et al.* Highly porous non-precious bimetallic electrocatalysts for efficient hydrogen evolution. *Nat. Commun.* **6**, 6567 (2015).
15. Gong, M. *et al.* Nanoscale nickel oxide/nickel heterostructures for active hydrogen evolution electrocatalysis. *Nat. Commun.* **5**, 4695 (2014).
16. Luo, J. *et al.* Water photolysis at 12.3% efficiency via perovskite photovoltaics and Earth-abundant catalysts. *Science* **345**, 1593–1596 (2014).
17. McKone, J. R., Sadtler, B. F., Werlang, C. A., Lewis, N. S. & Gray, H. B. Ni–Mo nanopowders for efficient electrochemical hydrogen evolution. *ACS Catal.* **3**, 166–169 (2013).
18. Lu, X. & Zhao, C. Electrodeposition of hierarchically structured three-dimensional nickel–iron electrodes for efficient oxygen evolution at high current densities. *Nat. Commun.* **6**, 6616 (2015).
19. Wang, H. *et al.* Bifunctional non-noble metal oxide nanoparticle electrocatalysts through lithium-induced conversion for overall water splitting. *Nat. Commun.* **6**, 7261 (2015).
20. Lu, Z. *et al.* Three-dimensional NiFe layered double hydroxide film for high-efficiency oxygen evolution reaction. *Chem. Commun.* **50**, 6479–6482 (2014).
21. Feng, L.-L. *et al.* High-index faceted Ni_3S_2 nanosheet arrays as highly active and ultrastable electrocatalysts for water splitting. *J. Am. Chem. Soc.* **137**, 14023–14026 (2015).

22. You, B. *et al.* High-performance overall water splitting electrocatalysts derived from cobalt-based metal–organic frameworks. *Chem. Mater.* **27**, 7636–7642 (2015).
23. Jiang, N., You, B., Sheng, M. & Sun, Y. Electrodeposited cobalt-phosphorous-derived films as competent bifunctional catalysts for overall water splitting. *Angew. Chem. Int. Ed.* **54**, 6251–6254 (2015).
24. Li, J. *et al.* Nanoparticle superlattices as efficient bifunctional electrocatalysts for water splitting. *J. Am. Chem. Soc.* **137**, 14305–14312 (2015).
25. Masa, J. *et al.* Amorphous cobalt boride (Co_2B) as a highly efficient nonprecious catalyst for electrochemical water splitting: oxygen and hydrogen evolution. *Adv. Energy Mater.* **6**, 1502313 (2016).
26. Hui, L. *et al.* Overall water splitting by graphdiyne-exfoliated and -sandwiched layered double-hydroxide nanosheet arrays. *Nat. Commun.* **9**, 5309 (2018).
27. Yu, F. *et al.* High-performance bifunctional porous non-noble metal phosphide catalyst for overall water splitting. *Nat. Commun.* **9**, 2551 (2018).
28. Kresse, G. & Furthmüller, J. Efficiency of ab-initio total energy calculations for metals and semiconductors using a plane-wave basis set. *Comput. Mater. Sci.* **6**, 15–50 (1996).
29. Kresse, G. & Joubert, D. From ultrasoft pseudopotentials to the projector augmented-wave method. *Phys. Rev. B* **59**, 1758 (1999).
30. Perdew, J. P., Burke, K. & Ernzerhof, M. Generalized gradient approximation made simple. *Phys. Rev. Lett.* **77**, 3865 (1996).
31. Rollmann, G., Rohrbach, A., Entel, P. & Hafner, J. First-principles calculation of the structure and magnetic phases of hematite. *Phys. Rev. B* **69**, 165107 (2004).
32. Hellman, F. *et al.* Interface-induced phenomena in magnetism. *Rev. Modern Phys.* **89**, 025006 (2017).
33. J. K. Nørskov *et al.*, Trends in the exchange current for hydrogen evolution. *J. Electrochem. Soc.* **152**, J23–J26 (2005).
34. J. Rossmeisl, A. Logadottir, J. K. Nørskov, Electrolysis of water on (oxidized) metal surfaces. *Chem. Phys.* **319**, 178–184 (2005).
35. McCrory, C. C. L., Jung, S. H., Peters, J. C. & Jaramillo, T. F. Benchmarking heterogeneous electrocatalysts for the oxygen evolution reaction. *J. Am. Chem. Soc.* **135**, 16977–16987 (2013).
